# Metformin Use and Cognitive Function in Older Adults With Type 2 Diabetes Following a Mediterranean Diet Intervention

**DOI:** 10.3389/fnut.2021.742586

**Published:** 2021-10-05

**Authors:** Natalia Soldevila-Domenech, Aida Cuenca-Royo, Nancy Babio, Laura Forcano, Stephanie Nishi, Cristina Vintró-Alcaraz, Carlos Gómez-Martínez, Susana Jiménez-Murcia, Rebeca Fernández-Carrión, Maria Gomis-González, Andrea Alvarez-Sala, Silvia Carlos, Xavier Pintó, Dolores Corella, Javier Díez-Espino, Olga Castañer, Fernando Fernández-Aranda, Jordi Salas-Salvadó, Rafael de la Torre

**Affiliations:** ^1^Integrative Pharmacology and Systems Neurosciences Research Group, Neurosciences Research Program, Hospital del Mar Medical Research Institute (IMIM), Barcelona, Spain; ^2^Department of Experimental and Health Sciences, Universitat Pompeu Fabra, Barcelona, Spain; ^3^Department of Biochemistry and Biotechnology, Hospital Universitari de Sant Joan de Reus, Institut d'Investigacions Sanitàries Pere i Virgili, Human Nutrition Unit, Universitat Rovira i Virgili, Reus, Spain; ^4^Centro de Investigación Biomédica en Red (CIBER) de Fisiopatología de la Obesidad y la Nutrición (CIBEROBN), Instituto de Salud Carlos III, Madrid, Spain; ^5^Department of Psychiatry, University Hospital of Bellvitge, Barcelona, Spain; ^6^Psychiatry and Mental Health Group, Neuroscience Program, Institut d'Investigació Biomèdica de Bellvitge (IDIBELL), L'Hospitalet de Llobregat, Barcelona, Spain; ^7^Department of Clinical Sciences, School of Medicine and Health Sciences, University of Barcelona, Barcelona, Spain; ^8^Department of Preventive Medicine, School of Medicine, University of Valencia, Valencia, Spain; ^9^Department of Preventive Medicine and Public Health, University of Navarra, Pamplona, Spain; ^10^Navarra Health Research Institute (IDISNA), Pamplona, Spain; ^11^Lipid Unit, Department of Internal Medicine, Institut d'Investigació Biomèdica de Bellvitge (IDIBELL), Hospital Universitari de Bellvitge, L'Hospitalet de Llobregat, Barcelona, Spain; ^12^Faculty of Medicine, Universitat de Barcelona, Barcelona, Spain; ^13^Cardiovascular Risk and Nutrition Research Group, Hospital del Mar Medical Research Institute (IMIM), Barcelona, Spain; ^14^Endocrinology Service, Hospital del Mar Medical Research Institute (IMIM), Barcelona, Spain

**Keywords:** cognition, Mediterranean diet, type 2 diabetes, metformin, metabolic syndrome, obesity, nutrition, overweight

## Abstract

**Background and Purpose:** Both adherence to the Mediterranean diet (MedDiet) and the use of metformin could benefit the cognitive performance of individuals with type 2 diabetes, but evidence is still controversial. We examined the association between metformin use and cognition in older adults with type 2 diabetes following a MedDiet intervention.

**Methods:** Prospective cohort study framed in the PREDIMED-Plus-*Cognition* sub-study. The PREDIMED-Plus clinical trial aims to compare the cardiovascular effect of two MedDiet interventions, with and without energy restriction, in individuals with overweight/obesity and metabolic syndrome. The present sub-study included 487 cognitively normal subjects (50.5% women, mean ± SD age of 65.2 ± 4.7 years), 30.4% of them (*N* = 148) with type 2 diabetes. A comprehensive battery of neurocognitive tests was administered at baseline and after 1 and 3 years. Individuals with type 2 diabetes that exhibited a good glycemic control trajectory, either using or not using metformin, were compared to one another and to individuals without diabetes using mixed-effects models with inverse probability of treatment weights.

**Results:** Most subjects with type 2 diabetes (83.1%) presented a good and stable glycemic control trajectory. Before engaging in the MedDiet intervention, subjects using metformin scored higher in executive functions (Cohen's *d* = 0.51), memory (Cohen's *d* = 0.38) and global cognition (Cohen's *d* = 0.48) than those not using metformin. However, these differences were not sustained during the 3 years of follow-up, as individuals not using metformin experienced greater improvements in memory (β = 0.38 vs. β = 0.10, *P* = 0.036), executive functions (β = 0.36 vs. β = 0.02, *P* = 0.005) and global cognition (β = 0.29 vs. β = −0.02, *P* = 0.001) that combined with a higher MedDiet adherence (12.6 vs. 11.5 points, *P* = 0.031). Finally, subjects without diabetes presented greater improvements in memory than subjects with diabetes irrespective of their exposure to metformin (β = 0.55 vs. β = 0.10, *P* < 0.001). However, subjects with diabetes not using metformin, compared to subjects without diabetes, presented greater improvements in executive functions (β = 0.33 vs. β = 0.08, *P* = 0.032) and displayed a higher MedDiet adherence (12.6 points vs. 11.6 points, *P* = 0.046).

**Conclusions:** Although both metformin and MedDiet interventions are good candidates for future cognitive decline preventive studies, a higher adherence to the MedDiet could even outweigh the potential neuroprotective effects of metformin in subjects with diabetes.

## Introduction

The prevalence of diabetes has reached epidemic proportions in the past few years. There are currently 463 million people living with diabetes, representing 8.5% of the world's adult population (1). It is estimated that diabetes affects 1 in 10 adults in Spain (1). Individuals with diabetes are at increased risk of blindness, lower limb amputation, and cardiovascular and kidney diseases, and have about 60% greater risk of developing dementia (2).

Diabetes consequences can be avoided or delayed with good glycemic control and the management of cardiovascular risk factors, which can be achieved by following a healthy diet, practicing regular physical activity, and smoking cessation (3). In individuals with type 2 diabetes, the traditional Mediterranean diet (MedDiet) has been shown to improve their glycemic control, various cardiovascular risk factors, and body weight (4, 5). In individuals without diabetes but with high cardiovascular risk, the MedDiet has also been shown to decrease the incidence of diabetes (6). Moreover, adherence to the MedDiet has been associated with improvements in some cognitive functions (7). Therefore, although type 2 diabetes has been associated with worse performance in executive functions (8), MedDiet interventions could be beneficial controlling glycemic levels and could prevent cognitive decline and even improve cognition in individuals with type 2 diabetes.

As lifestyle and weight management alone often fail to establish and sustain optimal glycemic control, glucose-lowering treatments are also an important component of diabetes management (3). Diabetes drugs may have indirect effects on the brain by affecting circulating concentrations of insulin and glucose (9, 10). However, although type 2 diabetes has been consistently associated with incident dementia, distinguishing between treated and untreated diabetes as a risk factor for dementia is challenging in most observational studies (11). Metformin has been used since the 1950s as first-line pharmacotherapy for treating patients with type 2 diabetes with good glycemic control because of its glucose-lowering effects, good safety profile and relatively low cost. However, for patients with poor glycemic control after this first-line therapy, alternative oral glucose-lowering medications and/or injectable insulin are preferable, in monotherapy or in combination with other therapy regimens (12).

Previous studies have shown that metformin use for more than 6 years was associated with lower risk of cognitive impairment (13) and with better performance in some cognitive domains over time in cognitively normal subjects with type 2 diabetes (14). However, there is currently considerable controversy about the effect of metformin on cognition (15). Given the varied response to glucose lowering medications and the heterogeneity in type 2 diabetes (16), the identification of different glycemic trajectory subgroups could help optimize the study of the effects of metformin on cognition. Thus, the analysis of trajectories of glycemic control is an important initial step toward the application of personalized medicine in the treatment of diabetes as it could help in the development of targeted strategies to improve the effectiveness of interventions (17).

The objective of this study is to characterize different glycemic trajectories subgroups and to examine the association between metformin use and cognition in subjects with type 2 diabetes that participated in the PREDIMED-Plus MedDiet intervention.

## Materials and Methods

### Study Design and Participants

The present study is a prospective cohort study framed in the PREDIMED-Plus-*Cognition* sub-study, using a subset of participants (*N* = 487) of the PREDIMED-Plus trial. Full details of the study design and procedures of the PREDIMED-Plus trial have been published elsewhere (5). Further details on the study inclusion/exclusion criteria as well as the study protocol are available at http://predimedplus.com/. Briefly, the PREDIMED-Plus study is an ongoing multi-center randomized parallel-group primary prevention trial (*N* = 6,874) designed to assess and compare the long-term effectiveness of an intensive lifestyle intervention with an energy-reduced Mediterranean diet (er-MedDiet, intervention group), physical activity (PA) promotion and behavioral support of weight loss goals, with a more common intervention featuring energy-unrestricted traditional MedDiet recommendations without any recommendations on PA and weight loss strategies (control group). In order to promote the adherence to the MedDiet both groups were free provided with an allotment of extra-virgin olive oil (1 L/month) and occasionally, tree nuts (125 g/month). Participant's recruitment took place between October 2013 and December 2016 across 23 Spanish hospitals, universities and research institutes. Participants were randomly assigned, in a 1:1 ratio, to intervention and control groups. The eligibility criteria for participants were community-dwelling adults with overweight grade II (18) or obesity [body mass index (BMI) between 27 and 40 kg/m^2^] from Primary Care Health Centers of the Spanish National Health System aged between 55 and 75 years in the case of men and between 60 and 75 years in women who met at least three criteria for metabolic syndrome. The clinical trial is registered at the International Standard Randomized Controlled Trials database (ISRCTN; 89898870).

Four study sites participated in the PREDIMED-Plus-*Cognition* sub-study, with an in-depth assessment of the cognitive performance at baseline, 1 and 3 years after the initiation of the assigned PREDIMED-Plus intervention: Hospital del Mar Medical Research Institute (IMIM), Barcelona; Pere Virgili Institute for Health Research (IISPV), Reus; University of Valencia (UV), Valencia; Bellvitge University Hospital (HUB), Hospitalet de Llobregat. Exclusion criteria for the present sub-study are included in Supplementary Table 1. The data were analyzed using the PREDIMED-Plus-*Cognition* database dated 14th January 2021. All participants gave written informed consent. The protocol of the PREDIMED-Plus-*Cognition* sub-study was approved by the local Research Ethics Committees from the participating centers and adheres to the standards of the World Medical Association (WAMA) Declaration of Helsinki.

### Outcomes and Assessments

#### Type 2 Diabetes

According to the American Diabetes Association criteria (19), type 2 diabetes was defined by previous clinical diagnosis of diabetes, HbA1c ≥ 6.5% (48 mmol/mol) or fasting plasma glucose >126 mg/dL at both the screening and baseline visit or use of oral anti-diabetic medication (metformin, dipeptidyl peptidase 4 inhibitors, sulfonylureas, insulin secretagogues, SLGT2 inhibitors or thiazolidinediones) or use of insulin.

#### Cognitive Performance

Cognitive function was assessed by trained neuropsychologists blinded to the participants' group assignment and included the following cognitive domains: (i) *Short-term and long-term auditory memory*, using the Rey's Auditory-Verbal Learning Test (RAVLT) (20); (ii) *Visuoconstructive praxis and attention, short- and long-term visuospatial memory and visual perception*, evaluated with the Rey–Osterrieth complex figure Test (RCFT) (21); (iii) *Processing speed*, evaluated with the Symbol Digit Modalities Test (SDMT) (22); (iv) *Inhibition and attention* (mental flexibility and interference resistance), evaluated with the Stroop Color-Word Interference Test (23); (v) *Decision-making abilities* (risk and reward and punishment values), evaluated with the Iowa Gambling Task (IGT) (24) (not administered to participants recruited at the UV site); (vi) *Inattentiveness, impulsivity, sustained attention and vigilance* evaluated with the Conners' Continuous Auditory Test of Attention (CPT) (25) (not administered to participants recruited at the UV site). Finally, a cognitive screening was also included at baseline using the Folstein Mini-Mental State Examination (MMSE) (26).

Composite scores of 3 cognitive domains, namely memory, executive function and global cognition, were calculated for each participant by standardizing raw test scores to z-scores using the mean and standard deviation of baseline data. The memory composite included the mean standardized individual scores of the RAVLT immediate and delayed scores and the RCFT immediate, delayed and recognition scores. The executive function composite included the RCFT copy score, the SDMT direct score, the Stroop interference score, the IGT total score and the CPT omission, commission and hit reaction time scores. Lastly, the global cognition composite included all the tests of memory and executive functions.

#### Depressive Symptomatology

The severity of depressive symptomatology was assessed using the Beck's Depression Inventory-II (BDI-II) (27) and was categorized according to general guidelines as no or minimal depression (0–9 points), mild-to-moderate depression (10–18 points), moderate-to-severe depression (19–29 points), and severe depression (≥30 points).

#### Anthropometry and Cardiovascular Biomarkers

Weight and height were measured by nurses using standardized procedures. BMI (kg/m^2^) was categorized as normo-weight (BMI 18.5–24.9 kg/m^2^), overweight (BMI 25.0–29.9 kg/m^2^), obesity I (BMI 30.0–34.9 kg/m^2^), and obesity II (BMI 35.0–39.9 kg/m^2^).

Fasting blood glucose, HbA1c and lipid levels (triglycerides, total cholesterol and HDL cholesterol) were determined using standard methodology after an overnight fast. LDL cholesterol concentrations were calculated using the Friedewald formula whenever triglycerides were lower than 300 mg/dL. Insulin was centrally measured by an electrochemiluminescence immunoassay using an Elecsys immunoanalyzer (Roche Diagnostics, Meylan, France). Insulin resistance was estimated at baseline using the Homeostasis Model Assessment of Insulin Resistance (HOMA-IR) index (28).

#### Intervention Adherence

Adherence to the er-MedDiet was evaluated with an adapted version of the validated 14-item PREDIMED questionnaire including 17-items, the energy-restricted Mediterranean Diet Adherence Screener (er-MEDAS) (29). Values ranged from 0 to 17 points and adherence was categorized as low (0–7 points), moderate (8–10 points), and high (11–17 points) using the cut-off values from previous studies based on approximate tertiles in the overall baseline PREDIMED-Plus sample (30). Physical activity categories (sedentary, under-active, moderately active, and active) were obtained from the Rapid Assessment of Physical Activity (RAPA) questionnaire (31).

#### Covariates

Covariates were evaluated at baseline through face-to-face interviews by trained staff using self-reported general questionnaires on socio-demographics (gender, age, years of education, employment status), lifestyle (smoking status), medication (use of treatment for high cholesterol, use of tranquilizers, or sedatives for anxiety or sleeping, use of medication for hypertension, use of medication for heart) and history of disease.

### Statistical Analyses

#### Identification of Latent HbA1c Trajectories Subgroups

Longitudinal finite mixture modeling was applied to explain the between-subject heterogeneity in growth of HbA1c by identifying latent classes or subgroups with different growth trajectories (32). Thus, each latent class represents a group of subjects sharing a similar HbA1c trajectory. We first applied latent class growth analysis (LCGA), and two different types of growth mixture modeling (GMM): GMM with random intercept and GMM with random intercept and slope. In each type of model, we tested 1–5 latent classes, so 15 models were computed in total. In order to select the model with the best or most reasonable representation of the observed data, our model selection criteria were based on lowest fit information criteria statistics, including the Bayesian Information Criterion (BIC), the Akaike Information Criterion (AIC) and the sample-size adjusted BIC (SABIC), as well as at high entropy. Entropy is a measure of classification uncertainty in class assignment, with higher values indicating clearer delineation of classes. All these models did not include any covariates. Once the best model was selected among this first set of 15 models, we compared it with 3 additional models that included covariates (intervention group, diabetes medications or time-by-group interaction) as predictors of the growth factors and the class. Following the previously mentioned criteria for model selection, the best model was finally selected. The selected model presented a good discrimination index, since subjects classified in class 1, 2, and 3 had a mean probability of 98.4, 89.9, and 93.2% of belonging to their class, respectively. See details in Supplementary Table 2.

#### Descriptive and Inferential Statistics

Descriptive statistics of study variables stratified by diabetes status (yes/no) and by diabetes subgroups were obtained as mean and standard deviation (SD) or 95% confidence intervals (95%CI) for continuous variables and percentages for categorical variables. Univariate differences were estimated with the unpaired *t* test for continuous variables and chi-square test or Fisher's exact test, as deemed appropriate, for categorical variables. Additionally, standardized mean differences between groups were computed as Cohen's d (abbreviated as “d”) with cut-offs for effect size interpretation of 0.2 (small), 0.5 (medium), 0.8 (large), and 1.2 (very large). Adjusted differences between diabetes subgroups at each time point (baseline, 1 and 3 years) were estimated with analysis of variance (ANOVA) from linear models adjusted by study site, years of education, use of treatment for high cholesterol, use of metformin and use of insulin. Correction for multiple comparisons was performed with the Tukey method when the explanatory variable was normally distributed and the Benjamini & Hochberg method otherwise. Finally, linear mixed effects models were used to test differences between groups in the mean rate of change in cognition from baseline, after 1 and 3 years.

#### Inverse Probability of Treatment Weights

Given that metformin was not randomly assigned, it was necessary to achieve comparability between groups with regard to pretreatment characteristics to reduce the potential confounding by indication bias and to get better estimates of the treatment effect. This was accomplished using inverse probability of treatment weights (IPTW) (33). IPTW are based on propensity scores estimated via generalized boosted models, a non-parametric machine-learning method that weights treated and control cases to estimate the population average treatment effect (ATE) weights. IPTW were used to generate a weighted “artificial” population (called “pseudo-population”) with almost perfect covariate balance, in which treatment and measured pretreatment characteristics are independent. Three different IPTW were computed for each one of the following comparisons: (i) individuals with type 2 diabetes treated with metformin vs. individuals with type 2 diabetes not treated with metformin, (ii) individuals with type 2 diabetes treated with metformin vs. individuals without type 2 diabetes, and (iii) individuals with type 2 diabetes not treated with metformin vs. individuals without type 2 diabetes. Absolute standardized difference in means or proportions (abbreviated as “D”), was used to evaluate comparability between groups before and after IPTW matching. The relative influence of each variable in the models was also reported and expressed as a percentage. When there were residual differences in pretreatment characteristics between groups in the matched sample, regression adjustment was used to control for those unbalanced factors, which is known as a doubly robust approach (33). Accordingly, the comparison between individuals with diabetes exposed and not exposed to metformin was adjusted by sleep apnea. The comparison between subjects with and without diabetes and subjects taking metformin was adjusted by years of education, smoking status, total cholesterol, LDL-cholesterol, and use of treatment for high cholesterol. All the subsequent analyses were performed using weighted regression with robust standard errors.

#### Missing Data and Software

Missing data was reported as absolute and relative frequencies (*N*, %) and each specific analysis was performed on individuals with complete information on the variables involved. We used the R package “twang” to compute IPTW, the package “survey” to compute the weighted analysis and the package “nlme” to estimate linear mixed effects models.

## Results

### Sample Characteristics

Baseline characteristics of study participants stratified by diabetes status are included in Table 1. Briefly, 50.5% were women, the mean (SD) age was 65.2 (4.7) years, 18.7% were employed and 62.1% were retired. Regarding their lifestyle, 12% were current smokers, most were underactive (66.9%) or sedentary (15.6%) and had a low or medium adherence to the er-MedDiet (45.4 and 41.5%, respectively). All participants were over-weight (27.3%) or obese (48.5% had obesity type I and 24.2% type II). Finally, 50.3% were taking medications for high cholesterol and 23.0% used tranquilizers or sedatives.

**Table 1:** Baseline characteristics of study participants stratified by type 2 diabetes (T2D) status and univariate differences.

		**All population**	**No-T2D**	**T2D**	*P* ^*^
**Variable**	**Category**	***N* (%)**	***N* (%)**	***N* (%)**	
***N***		487 (100)	339 (100)	148 (100)	
Study group	Intervention group	240 (49.3)	162 (47.8)	78 (52.7)	0.368
Study site	IMIM	116 (23.8)	65 (19.2)	51 (34.5)	** <0.001**
	IISPV	143 (29.4)	131 (38.6)	12 (8.1)	
	UV	70 (14.4)	34 (10.0)	36 (24.3)	
	HUB	158 (32.4)	109 (32.2)	49 (33.1)	
**Sociodemographic characteristics**					
Sex	Women	246 (50.5)	173 (51.0)	73 (49.3)	0.804
Age	*Mean (SD)*	65.2 (4.7)	64.9 (4.7)	65.9 (4.7)	**0.029**
Education (years)	*Mean (SD)*	11.7 (5.3)	12.1 (5.7)	10.5 (4.0)	** <0.001**
Employment status	Employed	91 (18.7)	68 (20.1)	23 (15.5)	0.611
	Unemployed	36 (7.4)	27 (8.0)	9 (6.1)	
	Housework	50 (10.3)	36 (10.7)	14 (9.5)	
	Retired	302 (62.1)	202 (59.8)	100 (67.6)	
	Missing	1	1		
**Lifestyle, obesity and mental health**					
Current smoker		59 (12.1)	47 (13.9)	12 (8.11)	0.101
Physical activity^a^	Sedentary	76 (15.6)	48 (14.2)	28 (18.9)	0.082
	Under-active	326 (66.9)	238 (70.2)	88 (59.5)	
	Moderately active	44 (9.03)	25 (7.37)	19 (12.8)	
	Active	41 (8.42)	28 (8.26)	13 (8.78)	
Er-MedDiet adherence^b^	Low	221 (45.4)	150 (44.2)	71 (48.0)	0.741
	Medium	202 (41.5)	144 (42.5)	58 (39.2)	
	High	64 (13.1)	45 (13.3)	19 (12.8)	
BMI category	Over-weight	133 (27.3)	99 (29.2)	34 (23.0)	0.156
	Obesity I	236 (48.5)	164 (48.4)	72 (48.6)	
	Obesity II	118 (24.2)	76 (22.4)	42 (28.4)	
Depressive symptomatology^c^	No or minimal	304 (62.4)	217 (64.0)	87 (58.8)	0.544
	Mild-to-moderate	140 (28.7)	93 (27.4)	47 (31.8)	
	Moderate-to-severe	43 (8.8)	29 (8.5)	14 (9.5)	
**Medications**					
Metformin		111 (22.7)	0 (0.00)	111 (75.0)	**-**
Insulin		10 (2.0)	0 (0.00)	10 (6.7)	**-**
Other treatments for diabetes^d^	51 (10.5)	0 (0.00)	51 (34.5)	**-**
Tranquilizers/sedatives	112 (23.0)	72 (21.2)	40 (27.0)	0.201
Cholesterol treatment	245 (50.3)	153 (45.1)	92 (62.2)	**0.001**
***Intelligence Quotient (PD)^e^***	*Mean (SD)*	92.0 (39.6)	88.7 (39.5)	99.5 (38.8)	0.006
**Glycemic profile**					
Hba1c (%)	*Mean (SD)*	6.1 (0.8)	5.8 (0.4)	7.0 (1.0)	** <0.001**
HbA1c (mmol/mol)	*Mean (SD)*	43.5 (9.2)	39.8 (4.2)	52.6 (11.2)	** <0.001**
Glucose (mg/dL)	*Mean (SD)*	116 (30.9)	103 (13.2)	146 (38.7)	** <0.001**
HOMA-IR index	*Mean (SD)*	5.6 (3.9)	5.0 (3.1)	7.1 (5.2)	** <0.001**

* *T-test for continuous variables [presented as mean (SD)]; and chi-squared test (or Fisher's exact test when expected count in some cells is lower than 5) for categorical variables*.

a *Obtained from the Rapid Assessment of Physical Activity (RAPA) questionnaire*.

b *Er-MedDiet, energy-restricted Mediterranean Diet adherence, from the 17-item er-MedDiet questionnaire*.

c *Obtained from the Beck's Depression Inventory-II (BDI)*.

d *Includes: dipeptidyl peptidase 4 inhibitors (N = 35), sulfonylureas (N = 21), insulin secretagogues (N = 11), SLGT2 inhibitors (N = 6), thiazolidinediones (N = 1), and others (N = 0)*.

e *Obtained from the WAIS-III Vocabulary Subtest*.

*HbA1c, glycosylated hemoglobin; IMIM, Hospital del Mar Medical Research Institute; IISPV, Pere Virgili Institute for Health Research; UV, University of Valencia; HUB, Bellvitge University Hospital. Bold values denote statistical significance at the p <0.05 level*.

Compared to individuals without diabetes, individuals with type 2 diabetes were older (65.9 vs. 64.9 years), had less years of education (10.5 vs. 12.1 years) and took more treatments for high cholesterol (62.2 vs. 45.1%). Moreover, most individuals with diabetes were being treated with metformin (75.0%), only 6.7% were taking insulin and 34.5% were taking other oral medications for diabetes (alone or in combination with metformin or insulin). As expected, participants with diabetes had poorer glycemic profile than those without diabetes, with higher values of HbA1c (mean of 7.0 vs. 5.8%), fasting plasma glucose (mean of 146 mg/dL vs. 103 mg/dL) and HOMA-IR index (mean of 5.0 vs. 7.1).

**Figure 1:**
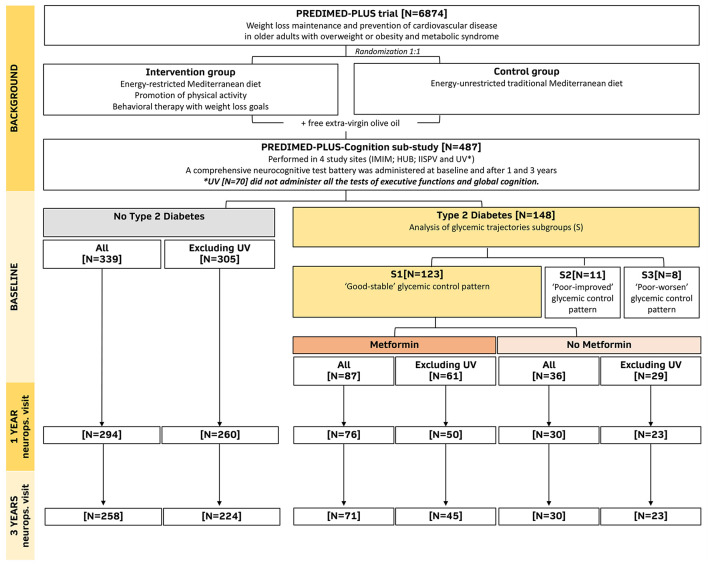
Study flow diagram including the follow-up in the neuropsychological visits (neurops. visit) after 1 and 3 years of intervention. IMIM, Hospital del Mar Medical Research Institute. IISPV, Pere Virgili Institute for Health Research. UV, University of Valencia. HUB, Bellvitge University Hospital.

Figure 1 includes a flow diagram of the follow-up of the present study.

### Longitudinal HbA1c Trajectories Subgroups

**Figure 2:**
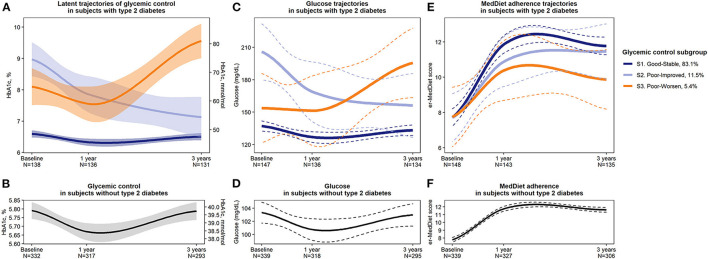
Representation of **(A)** glycemic control (HbA1c) latent trajectories or subgroups in subjects with diabetes, **(B)** HbA1c trajectory in subjects without diabetes, **(C)** fasting plasma glucose levels in each subgroup of subjects with diabetes and **(D)** in individuals without diabetes, and **(E)** er-MedDiet adherence score in diabetes subgroups and **(F)** in subjects without diabetes. Solid lines represent mean values, and shaded areas or dashed lines represent 95% confidence intervals (95% CI). HbA1c, glycosylated hemoglobin. *N*, number. S, subgroup.

First, participants with type 2 diabetes were classified into three distinct latent subgroups based on their HbA1c trajectory from baseline to 1 and 3 years of MedDiet intervention. Subgroup 1 (S1) contained most of the subjects with diabetes (83.1%, *N* = 123), and the remaining 11.5% (*N* = 17) and 5.4% (*N* = 8) were grouped into subgroups 2 (S2) and 3 (S3), respectively. Figure 2A shows the trajectories of HbA1c for the three different subgroups. At baseline, those in S1 presented good glycemic control (HbA1c <7%), with a mean (SD) of 6.6% (0.55), which decreased to 6.3% (0.6) after 1 year but following a return to 6.5% (0.7) after 3 years. This subgroup was termed “S1. Good-Stable glycemic control pattern.” S2 individuals presented poor glycemic control at baseline (HbA1c > 7%), with a mean (SD) of 9.0% (0.9), but it improved during the follow-up, with values of 7.9% (1.3) after 1 year and of 7.1% (1.1) after 3 years. This subgroup was termed “S2. Poor-Improved glycemic control pattern.” Finally, those in S3 also presented poor glycemic control at baseline with high HbA1c levels (mean of 8.1%, *SD* = 0.81). Although after 1 year their glycemic control slightly improved (HbA1c declined to a mean of 7.5%, *SD* = 0.70), after 3 years it worsened and increased to 9.6% (0.7). This subgroup was termed “S3. Poor-Worsen glycemic control pattern.” Figure 2B shows the HbA1c trajectory of individuals without diabetes.

As shown in Figure 2C, glucose trajectories in each subgroup mirrored HbA1c trajectories but with more variability, represented by a wider 95%CI. Figure 2D shows the mean glucose trajectory of individuals without diabetes. Regarding MedDiet adherence, at baseline, all subgroups scored a mean of 7.7–7.8 points, which is over the low adherence cut-off of 7 points and thus considered as moderate adherence. All subgroups presented mean improvements in their MedDiet adherence after 1 and 3 years. However, as shown in Figure 2E, S3 presented lower MedDiet adherence than S1 after 1 year (*d* = −0.87, 95% CI −1.59, −0.14) and especially after 3 years of follow-up (*d* = −1.16, 95% CI −1.89, −0.43). S2 also scored lower in MedDiet adherence than S1 after 1 year (*d* = −0.57, 95%CI −1.09, −0.04) but after 3 years they scored a high adherence for the MedDiet similar to the observed in S1 (11.5 vs. 11.8 points for S2 and S1, respectively). Despite of these moderate-to-large differences in MedDiet adherence between subgroups, results were not statistically significant, neither at baseline nor at the follow-up, probably due to the small subgroups sample size. See details in Supplementary Table 3. Figure 2F shows the MedDiet trajectory of individuals without diabetes.

Finally, metformin was used by 70.7% (*N* = 87) of participants from S1, and this treatment was maintained in 89.7% of them after 1 and 3 years of follow-up. From the remaining *N* = 36 participants from S1 that were not taking metformin at baseline, *N* = 26 (72.2%) continued without taking metformin throughout the 3 years of follow-up. Therefore, in S1 the metformin prescription did not change substantially throughout the follow-up (Supplementary Figure 1). Moreover, most of subjects from S1 who were not taking metformin did not take any treatment for type 2 diabetes (75%) or were taking other oral antidiabetic drugs (16.7%). The description and comparability between subgroups in terms of baseline characteristics and medications for diabetes is included in Supplementary Tables 4, 5.

### Metformin and Cognition in Subjects With Diabetes

Within the main subgroup of subjects with diabetes with a good-stable glycemic control (S1), those subjects using metformin (*N* = 87) were compared to those not using metformin (*N* = 36) (Supplementary Table 6). Before applying IPTW, those not using metformin presented higher total cholesterol (213 vs. 184 mg/dL) and higher LDL-cholesterol (131 vs. 108 mg/dL), but these differences vanished after matching with IPTW.

**Figure 3:**
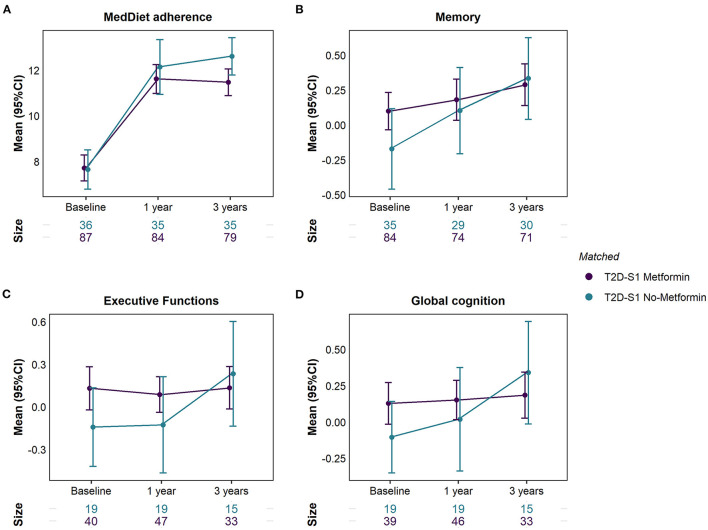
Differences in **(A)** MedDiet adherence, **(B)** Memory, **(C)** Executive functions, and **(D)** Global cognition between participants with type 2 diabetes from subgroup 1 (T2D-S1) treated and not treated with metformin (individuals were matched with inverse probability of treatment weights).

As shown in Table 2 and Figure 3, at baseline, individuals with type 2 diabetes from S1 treated with metformin scored moderately higher in memory (*d* = 0.38, 95% CI −0.02, 0.79; *P* = 0.115), executive functions (*d* = 0.51, 95% CI −0.06, 1.08; *P* = 0.086) and global cognition (*d* = 0.48, 95% CI −0.01, 1.04; *P* = 0.124) than those not treated with metformin. Nonetheless, an effect difference ranging from *d* = −0.06, a very small negative association, to 1.08, a large positive association, is also compatible with our data. However, no between-group differences in cognition were observed after 1 and 3 years of follow-up, given that those not treated with metformin compared to those treated with metformin experienced an improved performance from baseline to 3 years in memory (mean change of 0.38 vs. 0.10 SD from baseline mean [z-score], *P* = 0.036 for the between-group difference in mean change), executive functions (mean change of 0.36 vs. 0.02, *P* = 0.005) and global cognition (mean change of 0.29 vs. −0.02, *P* = 0.001).

**Table 2:** Differences in MedDiet adherence and in cognitive composites at each time point and in the mean change from baseline between individuals with type 2 diabetes from subgroup 1 (T2D-S1) treated with metformin and not treated with metformin (matched with inverse probability of treatment weights).

**Variable**	**Time**	**T2D-S1 No-Metformin**		**T2D-S1 Metformin**		**Differences**			**Differences in the mean change**		
		**[*****N*** **=** **36]^a^**		**[*****N*** **=** **87]^b^**		**at each time point**			**(95%CI) from baseline**		
		**Missing**	**Mean**	**Missing**	**Mean**	**Cohen's D**	**Effect**	***P* ^d^**	**No-Metformin**	**Metformin**	***P* ^e^**
		**[*N* (%)]**	**(95% CI)**	**[*N* (%)]**	**(95% CI)**	(95% CI)^*^	**Size^c^**			
er-MedDiet adherence score	Baseline	0 (0)	7.7 (6.8, 8.5)	0 (0)	7.7 (7.2, 8.3)	0.03 (−0.36, 0.41)	VS	0.752			
	1 year	1 (2.8)	12.1 (10.9, 13.3)	3 (3.4)	11.6 (11, 12.2)	−0.16 (−0.56, 0.23)	S	0.427	4.4 (3.4, 5.5)	3.9 (3.2, 4.6)	0.476
	3 years	1 (2.8)	12.6 (11.8, 13.4)	8 (9.2)	11.5 (10.9, 12.1)	−0.44 (−0.84, −0.02)	S	**0.031**	4.9 (3.9, 5.9)	3.9 (3.3, 4.4)	0.145
Memory composite (z-score)	Baseline	1 (2.8)	−0.17 (−0.46, 0.12)	3 (3.4)	0.1 (−0.03, 0.23)	0.38 (−0.02, 0.79)	S	0.115			
	1 year	7 (19.4)	0.1 (−0.21, 0.41)	13 (14.9)	0.18 (0.03, 0.33)	0.11 (−0.32, 0.54)	VS	0.795	0.2 (−0.03, 0.42)	0.01 (−0.11, 0.14)	0.307
	3 years	6 (16.7)	0.33 (0.04, 0.63)	16 (18.4)	0.29 (0.14, 0.44)	−0.06 (−0.49, 0.36)	VS	0.557	0.38 (0.15, 0.62)	0.1 (−0.05, 0.25)	**0.036**
Executive functions composite (*z*-score)^a^^,^ ^b^	Baseline	10 (34.5)	−0.14 (−0.42, 0.14)	21 (34.4)	0.13 (−0.02, 0.28)	0.51 (−0.06, 1.08)	M	0.086			
	1 year	10 (34.5)	−0.13 (−0.47, 0.21)	14 (23)	0.09 (−0.04, 0.21)	0.39 (−0.16, 0.93)	S	0.333	0.08 (0.00, 0.16)	−0.02 (−0.17, 0.13)	0.293
	3 years	14 (48.3)	0.23 (−0.14, 0.6)	28 (45.9)	0.14 (−0.01, 0.28)	−0.18 (−0.79, 0.44)	S	0.557	0.36 (0.13, 0.59)	0.02 (−0.09, 0.14)	**0.005**
Global cognition composite (*z*-score)^a^^,^ ^b^	Baseline	10 (34.5)	−0.1 (−0.35, 0.14)	22 (36.1)	0.13 (−0.02, 0.27)	0.48 (−0.1, 1.04)	M	0.124			
	1 year	10 (34.5)	0.02 (−0.34, 0.37)	15 (24.6)	0.15 (0.02, 0.29)	0.23 (−0.31, 0.77)	S	0.676	0.12 (−0.05, 0.29)	0.12 (0, 0.23)	0.511
	3 years	14 (48.3)	0.34 (−0.01, 0.69)	28 (45.9)	0.19 (0.03, 0.34)	−0.28 (−0.9, 0.34)	S	0.304	0.29 (0.10, 0.49)	−0.02 (−0.11, 0.07)	**0.001**

a *N = 29 and*

b *N = 61 when excluding participants from University of Valencia study site that did not receive all the tests from executive functions and global cognition*.

c *Effect Size: VS = very small (Cohen's d <0.2); S = small [Cohen's d (0.2–0.5)]; M = medium [Cohen's d (0.5–0.8)]; L = large [Cohen's d (0.8–1.2)]; VL = very large (Cohen's d ≥ 1.2)*.

d *ANOVA from multivariable-adjusted linear model*.

e *ANOVA from multivariable-adjusted linear mixed effects model*.

Inverse probability of treatment weighting (IPTW) was applied to all analyses to weight each individual with his/her inverse probability of being treated with metformin, generating a pseudo-population with (almost) perfect covariate balance. All models were adjusted by diagnosis of sleep apnoea.

* *Reference group= No-Metformin. Bold values denote statistical significance at the p <0.05 level*.

Supplementary Table 7 includes the differences in each specific cognitive test between subjects with type 2 diabetes (S1) treated and not treated with metformin. At baseline, those treated with metformin scored moderately higher in decision-making abilities (*d* = 0.60, *P* = 0.002) and visuoconstructive praxis and attention (*d* = 0.53, *P* = 0.018), as well as slightly higher in short- and long-term visual memory (*d* = 0.40, *P* = 0.050; and *d* = 0.45, *P* = 0.032, respectively). However, after 3 years, those using metformin scored lower in short- and long-term verbal memory (*d* = −0.42, *P* = 0.019; and *d* = −0.31, *P* = 0.051, respectively) and presented lower improvements in decision-making abilities (mean change of −1.2 vs. 15.9 points, *P* = 0.015).

Finally, at baseline, both groups presented a moderate adherence to the MedDiet (mean score of 7.7). However, after 3 years of dietary intervention, MedDiet adherence increased to 12.6 points (95% CI 11.8, 13.4) in subjects with type 2 diabetes not treated with metformin and to 11.5 points (95% CI 10.9, 12.1) in subjects treated with metformin, a difference which was statistically significant (*d* = −0.44, 95% CI −0.84, −0.02; *P* = 0.031).

### Diabetes vs. No Diabetes

Irrespective of metformin exposure, participants with type 2 diabetes from S1 were compared to participants without diabetes. Participants with diabetes presented lower total cholesterol, LDL-cholesterol, lower HDL-cholesterol and were more physically active (Supplementary Table 8). Despite large differences in the glycemic profile (Supplementary Table 9), both groups did not differ in terms of MedDiet adherence and BMI, although subjects with diabetes scored higher in depressive symptomatology at baseline (*d* = 0.39, 95% CI 0.18, 0.60) and after 1 year (*d* = 0.63, 95% CI 0.41, 0.85).

**Figure 4:**
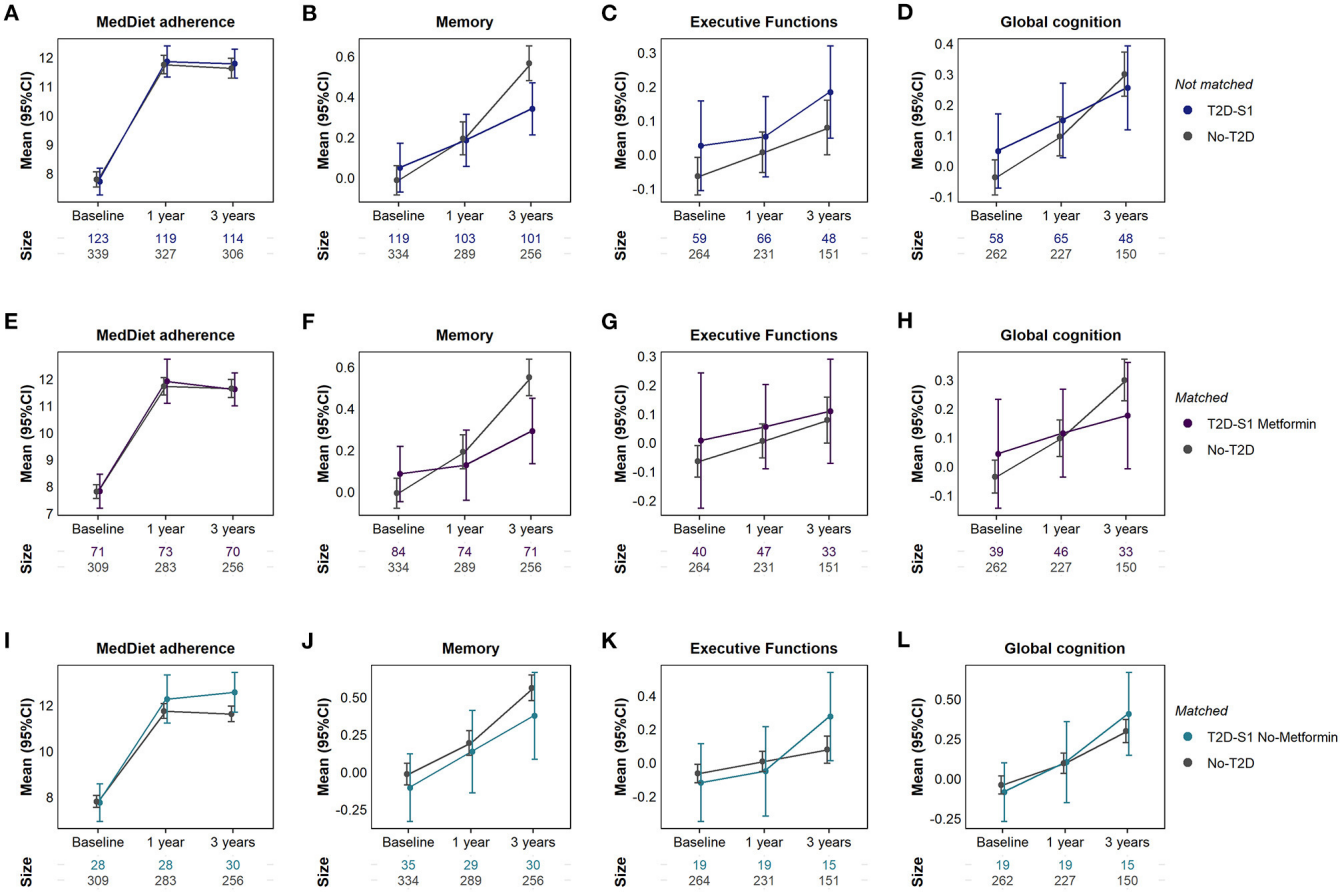
Differences in MedDiet adherence, memory, executive functions and global cognition between subjects without type 2 diabetes (No-T2D) and all subjects with type 2 diabetes from subgroup 1 (T2D-S1) **(A–D)**; T2D-S1 treated with metformin **(E–H)** and T2D-S1 not treated with metformin **(I–L)**. In comparisons **(E-L)** subjects with and without type 2 diabetes were matched with inverse probability of treatment weights.

There were no differences in baseline memory, executive functions and global cognition between subjects with and without type 2 diabetes (as shown in Figures 4A–D and Supplementary Table 10). However, from baseline to 3 years, those without diabetes exhibited a greater increase in their memory performance (mean change in memory z-score of 0.55 vs. 0.10, *P* < 0.001 for between group differences in mean change). This increase in the memory composite was mainly due to improvements in short- and long-term verbal memory. Subjects without diabetes also presented greater improvements in visuoconstructive praxis and attention (mean change of 2.8 vs. 0.9 points in RCFT figure copy task, *P* = 0.009) and did not present reductions in inhibition (mean change of −0.5 vs. −5.1 points in Stroop interference score), compared to subjects with diabetes. However, participants with diabetes presented greater reductions in the reaction time after 3 years (−48.5 ms vs. −0.5 ms in the CPT HRT, *P* = 0.016).

### Diabetes Plus Metformin vs. No Diabetes

Subjects with type 2 diabetes from S1 treated with metformin were compared to subjects without diabetes (Supplementary Table 11). In the matched analysis (Figures 4E–H and Supplementary Table 12), baseline cognition did not differ between individuals with diabetes treated with metformin and individuals without diabetes. However, subjects without diabetes showed greater increases in memory after 1 and 3 years (mean change in z-score of 0.53 vs. 0.14, *P* < 0.001) and in global cognition after 3 years (mean change in z-score of 0.25 vs. −0.001, *P* = 0.003), compared to subjects with diabetes treated with metformin. These two groups did not differ in terms of executive functions and MedDiet adherence, neither at baseline nor after 1 and 3 years of follow-up.

### Diabetes No-Metformin vs. No Diabetes

Participants without diabetes were compared to participants with type 2 diabetes from S1 who were not treated with metformin (Supplementary Table 13). Before matching these groups only differed in HDL-cholesterol (52.4 mg/dL in subjects without diabetes vs. 48.0 mg/dL in subjects with diabetes without metformin) but after matching this difference was balanced and no additional differences were observed. No differences in memory, executive functions and global cognition were observed between subjects with type 2 diabetes not treated with metformin and subjects without diabetes, except in the mean rate of change in executive functions, in which participants with diabetes not treated with metformin presented a greater improvement than participants without diabetes (mean change in z-score of 0.33 vs. 0.08, *P* = 0.032, as shown in Figures 4I–L and Supplementary Table 14). Moreover, participants with type 2 diabetes not treated with metformin also presented a greater adherence to the MedDiet after 3 years of follow-up (*d* = 0.32, 95%CI −0.03, 0.67; *P* = 0.046).

## Discussion

### Main Findings

This is the first study to date to examine the effect of metformin on cognition in older adults with type 2 diabetes following a MedDiet intervention. We first examined the heterogeneity in HbA1c trajectories after 1 and 3 years of dietary intervention. We identified three different subgroups of individuals with diabetes irrespective of the intervention group. The largest group exhibited good glycemic control that remained stable during the follow-up, while the remaining two subgroups showed poor baseline glycemic control that improved or worsened during the follow-up. Among the group with good glycemic control, we observed that those treated with metformin presented a better baseline performance in memory, executive functions and global cognition than those not treated with metformin. However, those not treated with metformin presented higher adherence to the MedDiet over time as well as greater improvements in memory, executive functions and global cognition, so that baseline differences between individuals with type 2 diabetes treated and not treated with metformin vanished after 1 and 3 years of MedDiet intervention. These results suggest that adherence to a MedDiet intervention could be superior to the potential neuroprotective effects of metformin among older adults with overweight/obesity and metabolic syndrome who have good glycemic control of their type 2 diabetes.

### Metformin Use and Cognition in Individuals With Diabetes

Our results suggest that metformin could have neuroprotective effects. Specifically, we observed that before starting the MedDiet intervention, individuals with type 2 diabetes from a group presenting good glycemic control (S1) treated with metformin presented a higher performance in memory, executive functions and global cognition than those not treated with metformin. These results agree with previous observational studies showing better memory performance (14) or greater maintenance of executive functions and global cognition (34) in cognitively normal subjects with diabetes type 2 treated with metformin, compared to those not treated with metformin. Metformin use has also been associated with lower dementia risk (35, 36) and better cognitive function (37), but results are still highly variable across studies (15). These inconsistencies could be explained by the fact that multiple neurocognitive pathways are affected by diabetes. Therefore, some pathways, but not all, may be improved with drug therapy (e.g., neurovascular alterations) (38). Moreover, metformin has the adverse effect of lowering serum vitamin B12 concentration, which can in turn, increase the risk of cognitive impairment (39).

Metformin mainly acts by reducing liver gluconeogenesis and inhibiting glucagon-mediated signaling in the liver, but it can also cross the blood brain barrier and thus affect the brain more directly (40). However, the potential neuroprotective effects of metformin have been mostly attributed to its anti-inflammatory and anti-coagulative properties, the prevention of metabolic syndrome (41) and the reduction of peripheral insulin levels that affect brain clearance of amyloid β-peptide (Aβ) (42). Several clinical trials have also tested the effects of metformin in subjects with mild cognitive impairment (MCI) (43) or Alzheimer's disease (AD) (40). In a pilot study in individuals with amnesic MCI (*N* = 80), metformin treatment for 12 months marginally improved the selective reminding score, but did not affect the global cognitive composite (ADAS-Cog) (43). However, in a cross-over RCT with 20 individuals with AD, metformin treatment for 8 weeks improved executive function and trends also suggested improved memory, learning and attention (40). These preliminary findings support the need for larger trials to evaluate the efficacy and cognitive safety of metformin in prodromal and dementia stages of AD, such as the one recently promoted by the University of Columbia (ClinicalTrials.gov Identifier: NCT04098666).

### Mediterranean Diet Adherence and Cognition in Individuals With Diabetes

Our results also suggest that a higher adherence to the MedDiet could reverse the cognitive disadvantage of those subjects with diabetes that were not treated with metformin, since both groups with diabetes achieved similar cognitive scores along the follow-up. Subjects with diabetes from S1 not treated with metformin presented improvements in memory, executive functions and global cognition composites during the 3 years of follow-up, but cognition remained almost stable among those treated with metformin. Moreover, subjects with diabetes who were not exposed to metformin showed greater adherence to the MedDiet after 3 years of follow-up. The reason for this is unknown, but this indicates a group of subjects with a high capacity to make lifestyle changes. In fact, prior to participating in this study, most subjects from this group were able to control their blood glucose without medication and when offered a lifestyle intervention they adhered to it very faithfully, which probably translated into cognitive improvement. Another possibility is that those individuals with type 2 diabetes who take anti-diabetic drugs value lifestyle interventions less than those who do not take any drugs. Moreover, the greater compliance with the MedDiet among individuals with type 2 diabetes not taking metformin at baseline may also explain why they did not require metformin during the 3 years of follow-up. Previous studies have already reported the delayed need of medication for diabetes in patients with a newly diagnosed type 2 diabetes after a MedDiet intervention, compared to a low-fat diet (44, 45).

The favorable effect of the MedDiet intervention was likely due to the overall composition of the dietary pattern and not to a decreased caloric intake, weight loss or increased physical activity, because the allocation to the intervention or control group was balanced among subjects with diabetes either treated and not treated with metformin. In individuals with type 2 diabetes, the MedDiet has been consistently associated with better glycemic control (reduction of HbA1c by 0.32–0.53%) and a better profile of cardiovascular risk factors, compared to low-fat diets (4). These mechanisms could explain why adherence to the MedDiet might improve cognition in individuals with type 2 diabetes (46, 47). The high content in plant-based foods of the MedDiet (olive oil, legumes, vegetables, fruit, cereals, and nuts), along with fish and moderate red wine consumption during meals, make the MedDiet rich in phenolic compounds, n-3 polyunsaturated fatty acids and vitamins that, in conjunction, may contribute to a reduced oxidative stress and chronic inflammation and better neurovascular health (48, 49).

### Differences Between Individuals With and Without Diabetes

When individuals with diabetes were compared to those without diabetes, we did not find baseline differences in memory, executive functions and global cognition composites. These results differ from previous cross-sectional studies in the overall PREDIMED-Plus population (*N* = 6,823) showing worse executive functioning (evaluated with different neuropsychological tests) at baseline among participants with type 2 diabetes (8). However, we observed that participants with diabetes experienced fewer improvements in memory than participants without diabetes after 3 years of follow-up.

Nevertheless, in our study subjects with diabetes not treated with metformin experienced a greater increase in their executive functions than subjects without diabetes after 3 years of follow-up. Therefore, their greater adherence to the MedDiet could explain this difference in the rate of change in executive functions. In turn, MedDiet adherence did not differ between subjects with diabetes treated with metformin and subjects without diabetes. However, those using metformin experienced a lower improvement in their memory after 1 and 3 years, and in their global cognition after 3 years of follow-up, compared to subjects without diabetes. Thus, in the face of equivalent adherence to MedDiet, metformin was unable to neutralize the negative impact of type 2 diabetes on cognition.

### Strengths

The strengths of this study include its longitudinal design with 3 years of follow-up and the large number of cognitive tests that are administered to participants, covering 12 different cognitive abilities that are grouped in memory, executive functions and global cognitive composites. Moreover, the methodology used in the analysis of results allowed us to minimize confounding by indication which is not frequently addressed in most studies of metformin and cognitive associations. Finally, we also described the heterogeneity in the response to a MedDiet intervention among individuals with diabetes type 2, which aligns with the current recommendations of more patient-centered research and care in the field of diabetes (3).

### Limitations

However, this study has several limitations. First, the small sample size of the population with diabetes (*N* = 148) leads to a small number of participants in the subgroups with poor glycemic control (*N* = 17 in S2 and *N* = 8 in S3). This limits the capacity to detect statistically significant differences in their baseline characteristics. Moreover, when testing the associations between metformin use and cognition within the group presenting good glycemic control (S1), the small sample size of the untreated (*N* = 36) and treated (*N* = 87) groups also limited the study of gender effects, which should be addressed in future studies. Moreover, this sample size constraint also prevented the stratification of subjects exposed and not exposed to metformin according to their adherence to the MedDiet (high/medium/low). Consequently, it was not possible to study the simultaneous effect (interaction) of metformin use and high MedDiet adherence on cognition.

Second, there were losses in the evaluation of the cognitive function during the follow-up (within S1, 14% in the first year and 18% in the third year). They were not unexpected given the burden of neuropsychological visits and the fact that visits of this sub-study were performed on different days to those of the main trial. In addition, executive functions and global cognition composites excluded participants from the UV study site (representing 27% of subjects from S1) since not all the tests that made up the construct of executive functions were administered in this site. Therefore, selection bias cannot be completely excluded from this study.

Third, our methodology was not suitable for investigating causal effects since metformin administration was not randomized, and we did not collect data on the duration of metformin use, specific doses, or patients' adherence to their medication regimens. However, we noted that participants did not change their metformin treatment during the 3 years of follow-up. Moreover, we used IPTW to match treated and untreated subjects in each comparison. This approach allows to account for systematic differences in comorbidities between groups and is used to limit confounding by indication. We also had no information about the *APOE* genotype of participants, which could influence the association between metformin use and cognitive decline, as reported in previous studies (14).

Finally, this study does not have a control group since all subjects were exposed to a MedDiet intervention. However, without any intervention, individuals with metabolic syndrome would have probably presented a cognitive decline over time (50) and in this study their cognition improved independently of their underlying pathological condition.

## Conclusions

In summary, both metformin and MedDiet seem to have neuroprotective effects in older adults at increased risk of pathological cognitive decline, presenting overweight/obesity, metabolic syndrome and type 2 diabetes. Given the heterogeneity in type 2 diabetes and in the response to lifestyle interventions and glucose-lowering medications, a group-based trajectory analysis was initially performed to stratify the population with diabetes. There were two minor subgroups with high HbA1c levels that did not achieve good glycemic control despite of the intensive MedDiet intervention. Future studies should consider applying more intensive and personalized dietary interventions to subjects with poor glycemic control of their type 2 diabetes. However, the majority subgroup of individuals with type 2 diabetes presented good glycemic control throughout the follow-up. In this subgroup, metformin treatment was associated with better memory, executive functions and global cognition at baseline. Nevertheless, after 1 and 3 years of MedDiet intervention, both metformin-treated and non-metformin-treated subjects achieved similar cognitive function. We postulate that increased adherence to the MedDiet explained the cognitive improvement observed in individuals with type 2 diabetes not treated with metformin. In conclusion, a high adherence to MedDiet seems to at least slow down cognitive decline in the elderly with metabolic syndrome and other chronic diseases. Our results support the hypothesis that both metformin and MedDiet interventions are good candidates for future cognitive decline preventive studies.

## Data Availability Statement

The datasets presented in this article are not readily available because there are restrictions on the availability of data for the PREDIMED-Plus trial, due to the signed consent agreements around data sharing. Requestors wishing to access the PREDIMED-Plus dataset generated and/or analyzed during the current study can make a request to the PREDIMED-Plus trial Steering Committee chair. Requests to access the datasets should be directed to Jordi Salas-Salvadó, jordi.salas@urv.cat.

## Ethics Statement

The studies involving human participants were reviewed and approved by Parc de Salut Mar Drug Research Ethics Committee, Clinical Research Ethics Committee of Bellvitge University Hospital, Drug Research Ethics Committee of the Institut d'Investigació Sanitària Pere Virgili and Committee of Ethics and Research on Humans of Valencia University. The patients/participants provided their written informed consent to participate in this study.

## Author Contributions

RT, NS-D, AC-R, and LF contributed to the conception and design of the study, wrote the manuscript, and reviewed/edited the manuscript. NS-D performed the statistical analyses. AC-R, LF, NB, SN, CG-M, RF-C, AA-S, CV-A, SJ-M, and OC contributed to data acquisition. DC, SC, JD-E, OC, MG-G, XP, JS-S, and FF-A contributed to critical revision of the manuscript for key intellectual content. RT, JS-S, and FF-A obtained funding for the study. All authors have read and approved the final manuscript.

## Funding

Study resulting from the following grants: SLT006/17/00246, SLT002/16/00045 and SLT006/17/00077 funded by the Department of Health of the Generalitat de Catalunya by the calls Acció instrumental de programes de recerca orientats en l'àmbit de la recerca i la innovació en salut and Pla estratègic de recerca i innovació en salut (PERIS). We thank CERCA Programme/Generalitat de Catalunya for institutional support. This project was funded by Instituto de Salud Carlos III (ISCIII), the Spanish Government Official Agency for funding biomedical research-with competitive grants leaded by JS-S and Josep Vidal for the periods 2014–2016, 2015–2017, 2017–2019, and 2018–2020, through the Fondo de Investigación para la Salud (FIS), which is co-funded by the European Regional Development Fund, ERDF, a way to build Europe) [grants: PI13/00233, PI13/00728, PI13/01123, PI13/00462, PI16/00533, PI16/00366, PI16/01094, PI16/00501, PI17/01167, PI19/00017, PI19/00781, PI19/01032, PI19/00576]; the Especial Action Project entitled: Implementación y evaluación de una intervención intensiva sobre la actividad física Cohorte PREDIMED-Plus grant to JS-S; the European Research Council [Advanced Research Grant 2014–2019; agreement #340918] granted to Miguel Ángel Martínez-González; the Recercaixa (number 2013ACUP00194) grant to JS-S. This research was also partially funded by EU-H2020 Grants (Eat2beNICE/H2020-SFS-2016-2; Ref 728018; and PRIME/H2020-SC1-BHC-2018-2020; Ref: 847879), Grant PROMETEO/2017/017 (Generalitat Valenciana) and Grant FEA/SEA 2017 for Primary Care Research. This work is also partially supported by ICREA under the ICREA Academia programme. This work was supported by grants from DIUE de la Generalitat de Catalunya 2017 SGR 138 from the Departament d'Economia i Coneixement de la Generalitat de Catalunya (Spain). NS-D has received the FI 2021 predoctoral grant (FI_B2021/00104) from the Agency for Management of University and Research Grants (AGAUR) of the Generalitat de Catalunya. CV-A was supported by a predoctoral Grant of the Ministerio de Educación, Cultura y Deporte (FPU16/01453). AA-S has received a post-doctoral grant from the Consellería de Innovación, Ciencia y Sociedad Digital, Generalitat Valenciana, Valencia (APOSTD/2020/003). The Physiopathology of Obesity and Nutrition Networking Biomedical Research Center (CIBEROBN) is an initiative of ISCIII.

None of these funding sources plays any role in the design, collection, analysis, or interpretation of the data or in the decision to submit manuscripts for publication. The funders of the study had no role in study design, data collection, data analysis, data interpretation, or writing of the report.

## Conflict of Interest

JS-S reports non-financial support from Nut and Dried Fruit Foundation, personal fees from Danone Institute Spain, other from Danone S.A., other from Font Vella Lanjaron, other from Nuts for Life, other from Eroski Distributors, outside the submitted work. FF-A reports consultation fees from Novo Nordisk and editor-in-chief honorarium from Wiley. JD-E reports honoraria for lectures and presentations from Novo Nordisk, Mundipharma, Lilly, Astra Zeneca, MSD and Boehringer Ingelheim. All these relationships did not influence study design, data collection, data analysis, data interpretation, or writing of the report. The remaining authors declare that the research was conducted in the absence of any commercial or financial relationships that could be construed as a potential conflict of interest.

## Publisher's Note

All claims expressed in this article are solely those of the authors and do not necessarily represent those of their affiliated organizations, or those of the publisher, the editors and the reviewers. Any product that may be evaluated in this article, or claim that may be made by its manufacturer, is not guaranteed or endorsed by the publisher.
