# Supplementary material for: Metformin Use and Cognitive Function in Older Adults With Type 2 Diabetes Following a Mediterranean Diet Intervention
Source: Front Nutr. 2021 Oct 5;8:742586. doi: 10.3389/fnut.2021.742586 (PMC8523839; doi:10.3389/fnut.2021.742586)

Supplementary Material

Table of contents

1 List of PREDIMED-Plus-*Cognition* sub-study investigators 3

2 Supplementary Tables 4

2.1 Supplementary Table 1. Exclusion criteria from the present study 4

2.2 Supplementary Table 2. Model fit statistics for latent classes of HbA1c in T2D subjects and selection of the best model (highlighted in grey) 5

2.3 Supplementary Table 3. Glycemic profile, MedDiet adherence, BMI and depressive symptomatology at baseline and after 1 and 3 years, according to the HbA1c latent subgroups of subjects with type 2 diabetes; and multivariable-adjusted differences 7

2.4 Supplementary Table 4. Characteristics of study participants with type 2 diabetes stratified by the latent subgroups of glycemic control (S1, S2, S3); and univariate differences 10

2.5 Supplementary Table 5. Diabetes pharmacotherapy pattern in each glycemic control subgroup of individuals with type 2 diabetes 12

2.6 Supplementary Table 6. Comparability of individuals with type 2 diabetes from subgroup 1 treated and not treated with metformin before and after matching with inverse probability of treatment weights (IPTW) 14

2.7 Supplementary Table 7. Differences in specific cognitive tests at each time point and in the mean change from baseline between individuals with type 2 diabetes from subgroup 1 (T2D-S1) treated with metformin and not treated with metformin (matched with inverse probability of treatment weights). 17

2.8 Supplementary Table 8. Comparability of subjects with type 2 diabetes from subgroup 1 (T2D-S1) and subjects without type 2 diabetes (No-T2D) 20

2.9 Supplementary Table 9. Glycemic profile, MedDiet adherence, BMI and depressive symptomatology at baseline and after 1 and 3 years in the population with type 2 diabetes from subgroup 1 (T2D-S1), compared to the population without type 2 diabetes (No-T2D); and multivariable-adjusted differences 22

2.10 Supplementary Table 10. Differences in MedDiet adherence and cognition at each time point and in the mean change from baseline between subjects without type 2 diabetes (No-T2D) and subjects with type 2 diabetes from subgroup 1 (T2D-S1). 24

2.11 Supplementary Table 11. Comparability between the population without type 2 diabetes (No-T2D) and the population with type 2 diabetes from subgroup 1 (T2D-S1) treated with Metformin before and after matching with inverse probability of treatment weights (IPTW) 27

2.12 Supplementary Table 12. Differences in MedDiet adherence and cognition at each time point and in the mean change from baseline between subjects without type 2 diabetes (No-T2D) and subjects with type 2 diabetes from subgroup 1 (T2D-S1, with 'Good-Stable' glycemic control

trajectory) treated with metformin (matched analyses with inverse probability of treatment weights) 30

2.13 Supplementary Table 13. Comparability of the population without type 2 diabetes (No-T2D) and the population with type 2 diabetes from subgroup 1 (T2D-S1) not treated with metformin before and after matching with inverse probability of treatment weights (IPTW).........33

2.14 Supplementary Table 14. Differences in MedDiet adherence and cognition at each time point and in the mean change from baseline between subjects without type 2 diabetes (No-T2D) and subjects with type 2 diabetes from subgroup 1 (T2D-S1, with 'Good-Stable' glycemic control trajectory) not treated with metformin (matched analyses with inverse probability of treatment weights) 36

3 Supplementary Figures 39

3.1 Supplementary Figure 1. Description of metformin treatment maintenance in subjects with type 2 diabetes from subgroup 1 ('Good Stable glycemic control pattern', N=123). 39

1 List of PREDIMED-Plus-*Cognition* sub-study investigators

Department of Preventive Medicine, University of Valencia, University Jaume I, Conselleria de Sanitat de la Generalitat Valenciana, Valencia, Spain: J.I. González, J.V. Sorlí, O. Portolés, R. Fernández-Carrión, C. Ortega-Azorín, R. Barragán, E.M. Asensio, O. Coltell, C. Sáiz, R. Osma, E. Férriz, I. González-Monje, F. Giménez-Fernández, L. Quiles, P. Carrasco, N. San Onofre, A. Carratalá-Calvo, C. Valero-Barceló, F. Antón, C. Mir, S. Sánchez-Navarro, J. Navas, I. González-Gallego, L. Bort-Llorca, L. Pérez-Ollero, M. Giner-Valero, R. Monfort-Sáez, J. Nadal-Sayol, V. Pascual-Fuster, M. Martínez-Pérez, C. Riera, M.V. Belda, A. Medina, E. Miralles, M.J. Ramírez-Esplugues, M. Rojo-Furió, G. Mattingley, M.A. Delgado, M.A. Pages, Y. Riofrío, L. Abuomar, N. Blasco-Lafarga, R. Tosca, L. Lizán, A.M. Valcarce, M.D. Medina, R. Monfort, S. de Valcárcel, N. Tormo, O. Felipe-Román, S. Lafuente, E.I. Navío, G. Aldana, J.V. Crespo, J.L. Llosa, L. González-García, R. Raga-Marí.

Rovira i Virgili University, Department of Biochemistry and Biotechnology, Human Nutrition Unit, University Hospital of Sant Joan de Reus, Pere Virgili Institute for Health Research, Reus, Spain: R. Pedret Llaberia, R. Gonzalez, R. Sagarra Álamo, F. París Palleja, J. Balsells, J.M. Roca, T. Basora Gallisa, J. Vizcaino, P. Llobet Alpizarte, C. Anguera Perpiñá, M. Llauradó Vernet, C. Caballero, M. Garcia Barco, M.D. Morán Martínez, J. García Rosselló, A. Del Pozo, C. Poblet Calaf, P. Arcelin Zabal, X. Floresví, M. Ciutat Benet, A. Palau Galindo, J.J. Cabré Vila, F. Dolz Andrés, M. Soler, M. Gracia Vidal, J. Vilalta J. Boj Casajuana, M. Ricard, F. Saiz, A. Isach, M. Sanchez Marin Martinez, E. Granado Font, C, Lucena Luque, M. Bulló, N. Babio, N. Becerra-Tomás, G. Mestres, J. Basora, G. Mena-Sánchez, L. Barrubés Piñol, M. Gil Segura, C. Papandreou, N. Rosique-Esteban, S. Chig, I. Abellán Cano, V. Ruiz García, A. Salas-Huetos, I. Paz-Graniel, L. Sánchez Niembro, P. Hernandez-Alonso, S. Canudas, L. Camacho-Barcia, J. García-Gavilán, A. Díaz-López.

Cardiovascular Risk and Nutrition Research Group, Servicio de Endocrinología, IMIM (Hospital del Mar Medical Research Institute), Barcelona. Departament de Medicina, Universitat Autònoma de Barcelona, Barcelona, Spain: M. Fitó, O. Castañer, M.A. Muñoz, M.D. Zomeño, A. Hernaéz, L. Torres, M. Quifer, R. Llimona, L.A. Gal, A. Pérez, M. Farràs, R. Elosua, J. Marrugat, J. Vila, I. Subirana, S. Pérez, M.A. Muñoz, A. Goday, J.J. Chillaron Jordan, J.A. Flores Lerroux, D. Benaiges Boix, D. Muñoz-Aguayo, M. Gomis, S. Gaixas, G. Blanchart, A. Sanllorente, M. Soria, J. Valussi, A. Cuenca, L. Forcano, A. Pastor, A. Boronat, A. Aldea, A., M Perez, S Tello, M. Cabañero, L. Franco, H. Schröder, R. Medrano, J. Bayó, M.T. García, V. Babi, E. Canals, N. Soldevila, L. Carrés, C. Roca, M.S. Comas, G. Gasulla, X. Herraiz, A. Martínez, E. Vinyoles, J.M. Verdú, M. Masague Aguade, E. Baltasar Massip, M. Lopez Grau, M. Mengual, V. Moldon, M. Vila Vergaz, R. Cabanes Gómez, Ciurana, M. Gili Riu, A. Palomeras Vidal.

Department of Psychiatry, University Hospital of Bellvitge, Barcelona, Spain: F. Fernández-Aranda, S. Jiménez-Murcia, N. Mallorqui-Bagué, R. Granero, Z. Agüera, M. Lozano-Madrid.

2 Supplementary Tables

2.1 Supplementary Table 1. Exclusion criteria from the present study

Number	Criteria
1	History of chronic medical illness or neurological condition that may affect cognitive function
2	Current psychiatric diagnosis or in the year prior to inclusion
3	Traumatic brain injury with loss of consciousness more than 2 minutes, learning disorder, mental retardation or psychotic disorder
4	Psychoactive substance abuse or dependence (either currently or in the past 6 months)
5	Comorbid Eating Disorder (DSM -IV-TR criteria; APA, 2000)

2.2 Supplementary Table 2. Model fit statistics for latent classes of HbA1c in T2D subjects and selection of the best model (highlighted in grey)

Models		Model Nº	Nº Classes	Free Param.	AIC	BIC	SABIC	Entropy	Class 1 (%)	Class 2 (%)	Class 3 (%)	Class 4 (%)	Class 5 (%)
**Step 1**	LCGA (No within-class variance)	1	1	3	1153.8	1162.8	1153.3	1.00	100	-	-	-	-
		2	2	6	1033.9	1051.9	1032.9	0.92	87.8	12.2	-	-	-
		3	3	9	999.0	1026.0	997.5	0.75	41.2	9.5	49.3	-	-
		4	4	12	983.5	1019.5	981.5	0.78	41.9	8.1	43.2	6.8	-
		5	5	15	950.4	995.4	947.9	0.84	4.7	7.4	42.6	2.7	42.6
	GMM with random intercept	6	1	4	1014.0	1026.0	1013.3	1.00	100	-	-	-	-
		7	2	8	994.0	1017.9	992.6	0.88	9.5	90.5	-	-	-
		8	3	12	943.8	979.8	941.8	0.92	5.4	84.5	10.1	-	-
		9	4	16	944.6	992.6	941.9	0.77	51.4	33.1	5.4	10.1	-
		10	5	20	951.7	1011.7	948.4	0.71	16.9	5.4	33.1	33.8	10.8
	GMM with random intercept and slope	11	1	6	1016.1	1034.1	1015.1	1.00	100	-	-	-	-
		12	2	10	971.2	1001.2	969.6	0.62	18.2	81.8	-	-	-
		**13**	**3**	**14**	**945.1**	**987.0**	**942.7**	**0.93**	**83.1**	**11.5**	**5.4**	**-**	**-**
		14	4	18	946.9	1000.8	943.9	0.78	33.8	11.5	5.4	49.3	-
		15	5	22	958.3	1024.2	954.6	0.68	33.1	8.1	33.8	15.5	9.5
**Step 2**	**Comparison of Model #13 (without covariates) with:**												
	Model 13 + Intervention group as covariate	13.1	3	17	946.4	997.4	943.6	0.93	5.4	83.8	10.8	-	-
	Model 13 + Intervention group & diabetes medications as covariates	13.2	3	26	918.2	996.1	913.8	0.70	46.6	14.9	38.5	-	-

Model 13 + Time-by-group interaction as covariate	13.3	3	20	953.8	1013.7	950.4	0.72	35.8	17.6	46.6	-	-

LCGA= latent class growth analysis. It is a special type of GMM, whereby the variance and covariance estimates for the growth factors within each class are assumed to be fixed to zero. By this assumption, all individual growth trajectories within a class are homogeneous.

GMM= growth mixture model. It allows for differences in growth parameters across unobserved subpopulations, so different groups of individual growth trajectories can vary around different means (intercept) and forms (slope).

First, models were compared in terms of relative information criteria, in order to select those with the lowest information criteria. Model #8 (3-class GMM with random intercept) had the lowest BIC (BIC=979.8). However, model #13 (3-class GMM with random intercept and slope) presented a BIC of 987.0, so it differed in <10 points from Model #8 and had equivalent AIC and SABIC values. Moreover, both presented high accuracy, with entropy values of 0.92 and 0.93 for Model #8 and #13, respectively. Entropy is an indicator of the conditional probabilities of individuals' group membership, so high values >0.80 indicate that individuals are classified with confidence and there is adequate separation between the latent classes. Therefore, model #13 was selected as the best one among this first set of models. Then, in a second step, model #13 was compared to equivalent models that included covariates (intervention group, medications or time-by-group interaction) as predictors of the growth factors and the class (labeled as models #13.1, #13.2 and #13.3). However, model #13 without covariates remained the one with the lowest BIC and the highest entropy, and it was finally selected as the best model for studying the latent classes of HbA1c.

BIC= Bayesian Information Criteria. AIC= Akaike Information Criterion. SABIC= sample-size adjusted BIC.

2.3 Supplementary Table 3. Glycemic profile, MedDiet adherence, BMI and depressive symptomatology at baseline and after 1 and 3 years, according to the HbA1c latent subgroups of subjects with type 2 diabetes; and multivariable-adjusted differences

Variable	Time	Sub-group	Missing N (%)	Mean (95%CI)	Difference between subgroups									
					Effect size difference[1]		Model 1 (unadjusted) P-values				Model 2 (adjusted)[2] P-values			
					Cohen's D (95%CI)	Size	Overall	S1 *vs* S2	S1 *vs* S3	S2 *vs* S3	Overall	S1 *vs* S2	S1 *vs* S3	S2 *vs* S3
Glycosylated hemoglobin, HbA1c (%)	Baseline	S1	9 (7.3)	6.6 (6.5, 6.7)	Ref		**<0.001**	**<0.001**	**<0.001**	**0.001**	**<0.001**	**<0.001**	**<0.001**	**0.001**
		S2	0 (0.0)	9.0 (8.5, 9.4)	3.08 (2.43, 3.72)	VL								
		S3	1 (12.5)	8.1 (7.3, 8.9)	2.00 (1.19, 2.80)	VL								
	1 year	S1	10 (8.1)	6.3 (6.2, 6.4)	Ref		**<0.001**	**<0.001**	**0.013**	0.193	**<0.001**	**<0.001**	**0.013**	0.193
		S2	2 (11.8)	7.9 (7.1, 8.6)	1.80 (1.21, 2.39)	VL								
		S3	0 (0.0)	7.6 (7, 8.1)	1.52 (0.77, 2.26)	VL								
	3 years	S1	12 (9.8)	6.5 (6.4, 6.6)	Ref		**<0.001**	**0.048**	**<0.001**	**<0.001**	**<0.001**	**0.048**	**<0.001**	**<0.001**
		S2	5 (29.4)	7.1 (6.4, 7.8)	0.75 (0.14, 1.35)	M								
		S3	0 (0.0)	9.6 (9, 10.2)	3.76 (2.88, 4.62)	VL								
Fasting plasma glucose (mg/dL)	Baseline	S1	1 (0.8)	137.1 (132.5, 141.8)	Ref		**<0.001**	**<0.001**	0.350	**0.003**	**<0.001**	**<0.001**	0.350	**0.003**
		S2	0 (0.0)	205.9 (178.4, 233.4)	12.74 (11.03, 14.39)	VL								
		S3	0 (0.0)	153.8 (108.7, 198.8)	3.17 (2.35, 3.99)	VL								
	1 year	S1	10 (8.1)	126.9 (122.1, 131.8)	Ref		**<0.001**	**<0.001**	0.091	0.647	**<0.001**	**<0.001**	0.091	0.647
		S2	2 (11.8)	169.0 (141.2, 196.8)	7.87 (6.71, 9.03)	VL								
		S3	0 (0.0)	151.4 (120, 182.8)	4.75 (3.79, 5.69)	VL								
	3 years	S1	10 (8.1)	133.2 (128.1, 138.4)	Ref		**<0.001**	0.061	**<0.001**	**0.012**	**<0.001**	0.061	**<0.001**	**0.012**

		S2	4 (23.5)	156.2 (122.9, 189.4)	4.17 (3.37, 4.95)	VL									
		S3	0 (0.0)	195.6 (164.2, 227.0)	11.75 (10.05, 13.44)	VL									
er-MedDiet adherence	Baseline	S1	0 (0.0)	7.7 (7.3, 8.2)	Ref		0.999	0.948	0.995	0.961	0.999	0.948	0.995	0.961
		S2	0 (0.0)	7.7 (6.4, 9.0)	-0.01 (-0.52, 0.50)	VS									
		S3	0 (0.0)	7.8 (6.2, 9.3)	0.02 (-0.7, 0.73)	VS									
	1 year	S1	4 (3.3)	11.8 (11.3, 12.4)	Ref		0.201	0.532	0.401	0.867	0.206	0.532	0.401	0.867
		S2	1 (5.9)	10.9 (9.3, 12.4)	-0.57 (-1.09, -0.04)	M									
		S3	0 (0.0)	10.4 (8.5, 12.3)	-0.87 (-1.59, -0.14)	L									
	3 years	S1	9 (7.3)	11.8 (11.3, 12.3)	Ref		0.161	0.999	0.323	0.404	0.162	0.999	0.323	0.404
		S2	4 (23.5)	11.5 (9.6, 13.3)	-0.19 (-0.76, 0.39)	S									
		S3	0 (0.0)	9.9 (7.6, 12.1)	-1.16 (-1.89, -0.43)	VL									
BMI (kg/m^2)	Baseline	S1	0 (0.0)	32.8 (32.2, 33.4)	Ref		0.490	0.744	0.772	0.549	0.492	0.744	0.772	0.549
		S2	0 (0.0)	33.8 (32.2, 35.4)	0.56 (0.05, 1.07)	M									
		S3	0 (0.0)	32.6 (29.3, 35.9)	-0.09 (-0.81, 0.62)	VS									
	1 year	S1	1 (0.8)	31.3 (30.6, 31.9)	Ref		0.171	0.345	0.848	0.392	0.172	0.345	0.848	0.392
		S2	0 (0.0)	33.0 (31.3, 34.6)	0.91 (0.39, 1.43)	L									
		S3	0 (0.0)	31.5 (28.4, 34.7)	0.14 (-0.57, 0.86)	VS									
	3 years	S1	4 (3.3)	31.3 (30.7, 32.0)	Ref		0.366	0.565	0.888	0.562	0.370	0.565	0.888	0.562
		S2	1 (5.9)	32.7 (30.7, 34.6)	0.72 (0.19, 1.25)	M									
		S3	0 (0.0)	31.7 (27.9, 35.4)	0.18 (-0.53, 0.90)	S									
Depressive symptomatology (BDI-II total	Baseline	S1	0 (0.0)	9.2 (7.9, 10.4)	Ref		0.855	0.920	0.533	0.460	0.851	0.920	0.533	0.460
		S2	0 (0.0)	10.2 (5.7, 14.8)	0.39 (-0.12, 0.90)	S									

score)		S3	0 (0.0)	9.6 (1.5, 17.8)	0.17 (-0.55, 0.88)	S								
	1 year	S1	10 (8.1)	7.4 (6.2, 8.6)	Ref		0.332	0.435	0.904	0.923	0.340	0.435	0.904	0.923
		S2	2 (11.8)	9.9 (5.3, 14.5)	0.96 (0.41, 1.52)	L								
		S3	0 (0.0)	9.8 (-0.6, 20.1)	0.88 (0.16, 1.61)	L								
	3 years	S1	12 (9.8)	6.2 (5.0, 7.5)	Ref		0.485	0.911	0.557	0.783	0.484	0.911	0.557	0.783
		S2	4 (23.5)	6.8 (3.8, 9.9)	0.24 (-0.34, 0.81)	S								
		S3	1 (12.5)	9.3 (1.6, 17.0)	1.17 (0.39, 1.95)	VL								

[1]Effect Size: VS= very small (Cohen's d <0.2); S= small (Cohen's d (0.2-0.5)); M= medium (Cohen's d (0.5-0.8)); L= large (Cohen's d (0.8-1.2)); VL= very large (Cohen's d ≥ 1.2).

[2]Adjusted by study site, years of education, use of treatment for cholesterol, use of metformin and use of insulin. Correction for multiple comparisons was performed with the Tukey HSD (Honestly-significant-difference) test.

er-MedDiet= energy-restricted MedDiet adherence (from the adapted 17-item questionnaire). BDI=Beck's Depression Inventory -II. BMI= body mass index. HbA1c= glycosylated hemoglobin. 95%CI= 95% confidence interval. Ref=reference group.

S1= subgroup with a 'good-stable' glycemic control pattern. S2= subgroup with a 'poor-improved' glycemic control pattern. S3= subgroup with 'poor-worsen' glycemic control pattern.

2.4 Supplementary Table 4. Characteristics of study participants with type 2 diabetes stratified by the latent subgroups of glycemic control (S1, S2, S3); and univariate differences

Variable	Category	S1<br>N (%)	S2<br>N (%)	S3<br>N (%)	Overall difference<br>P-value	S1 *vs* S2<br>P-value	S1 *vs* S3<br>P-value	S2 *vs* S3<br>P-value
**N**		123 (100)	17 (100)	8 (100)				
Study group	Intervention group	69 (56.1)	5 (29.4)	4 (50.0)	0.125	0.212	0.732	0.591
Study site	IMIM	41 (33.3)	7 (41.2)	3 (37.5)	0.458	0.705	0.959	0.999
	IISPV	12 (9.76)	0 (0.0)	0 (0.0)				
	UV	33 (26.8)	2 (11.8)	1 (12.5)				
	HUB	37 (30.1)	8 (47.1)	4 (50.0)				
Sociodemographic characteristics								
Sex	Women	63 (51.2)	7 (41.2)	3 (37.5)	0.594	0.907	0.907	0.999
Age	*Mean (SD)*	66.5 (4.6)	63.5 (4.6)	63.1 (5.2)	**0.012**	**0.042**	0.123	0.977
Education (years)	*Mean (SD)*	10.3 (3.96)	11.5 (4.60)	11.9 (4.12)	0.317	0.471	0.537	0.978
Employment status	Employed	15 (12.2)	4 (23.5)	4 (50.0)	0.099	0.597	0.12	0.722
	Unemployed	6 (4.9)	2 (11.8)	1 (12.5)				
	Housework	13 (10.6)	1 (5.88)	0 (0.0)				
	Retired	87 (70.7)	10 (58.8)	3 (37.5)				
	Other	2 (1.6)	0 (0.0)	0 (0.0)				
	Missing	0	0	0				
Lifestyle, Obesity and Mental Health								
Current smoker		9 (7.32)	3 (17.6)	0 (0.00)	0.259	0.49	0.999	0.79
BMI category	Over-weight	30 (24.4)	2 (11.8)	2 (25.0)	0.682	0.651	0.651	0.651
	Obesity I	58 (47.2)	9 (52.9)	5 (62.5)				
	Obesity II	35 (28.5)	6 (35.3)	1 (12.5)				
Physical activity[1]	Sedentary	21 (17.1)	3 (17.6)	4 (50.0)	0.178	0.306	0.306	0.306
	Under-active	71 (57.7)	13 (76.5)	4 (50.0)				
	Moderately active	19 (15.4)	0 (0.0)	0 (0.0)				
	Active	12 (9.8)	1 (5.9)	0 (0.0)				

Er-MedDiet adherence[2]	High	17 (13.8)	2 (11.8)	0 (0.0)	0.620	0.699	0.699	0.699
	Medium	45 (36.6)	9 (52.9)	4 (50.0)				
	Low	61 (49.6)	6 (35.3)	4 (50.0)				
Depressive symptomatology[3]	No or Minimal	72 (58.5)	9 (52.9)	6 (75.0)	0.448	0.692	0.692	0.72
	Mild-to-moderate	41 (33.3)	5 (29.4)	1 (12.5)				
	Moderate-to-severe	10 (8.13)	3 (17.6)	1 (12.5)				
Medications								
Tranquilizers/sedatives at baseline		35 (28.5)	3 (17.6)	2 (25.0)	0.756	0.999	0.999	0.999
Cholesterol treatment at baseline		75 (61.0)	11 (64.7)	6 (75.0)	0.883	0.999	0.999	0.999
Metformin	Baseline	87 (70.7)	17 (100)	7 (87.5)	0.075	0.128	0.66	0.999
	Year 1	77 (62.6)	17 (100)	5 (62.5)	**0.019**	0.063	1	0.121
	Year 3	83 (68.0)	16 (94.1)	7 (87.5)	**0.041**	0.073	0.65	1
	Missing	1	0	0				
Insulin	Baseline	5 (4.07)	1 (5.88)	4 (50.0)	**0.001**	0.547	**0.002**	**0.035**
	Year 1	5 (4.07)	2 (11.8)	4 (50.0)	**0.001**	0.202	**0.002**	0.089
	Year 3	5 (4.13)	7 (41.2)	5 (62.5)	**<0.001**	**<0.001**	**<0.001**	0.411
	Missing	2	0	0				
Other treatments for diabetes[4]	Baseline	34 (27.6)	13 (76.5)	4 (50.0)	**<0.001**	**0.001**	0.343	0.359
	Year 1	39 (31.7)	12 (70.6)	4 (50.0)	**0.005**	**0.013**	0.438	0.438
	Year 3	42 (35.3)	13 (76.5)	5 (62.5)	**0.002**	**0.009**	0.217	0.640
	Missing	4	0	0				
Intelligence Quotient (PD)[5]		98.4 (40.5)	99.8 (30.3)	115 (24.8)	0.508	0.99	0.475	0.634

*T-test for continuous variables (presented as mean (SD)); and chi-squared test (or exact Fisher test when expected count in some cells is lower than 5) for categorical variables. Correction for multiple comparisons was performed with the Tukey method when explanatory variable was normal-distributed and Benjamini & Hochberg method otherwise.

[1]Obtained from the Rapid Assessment of Physical Activity (RAPA) questionnaire.

[2]Er-MedDiet, energy-restricted Mediterranean Diet adherence, from the 17-item er-MedDiet questionnaire.

[3]Obtained from the Beck's Depression Inventory-II (BDI).

[4]Includes: dipeptidyl peptidase 4 inhibitors, sulfonylureas, insulin secretagogues, SLGT2 inhibitors, thiazolidinediones and others (Glucagon-like peptide-1 analogs, alpha glycosidase

inhibitors, other biguanide medication and other hypoglycemic drugs).

[5]Obtained from the WAIS-III Vocabulary Subtest.

S1= subgroup with a 'good-stable' glycemic control pattern. S2= subgroup with a 'poor-improved' glycemic control pattern. S3= subgroup with 'poor-decline' glycemic control pattern. IMIM= Hospital del Mar Medical Research Institute. IISPV= Pere Virgili Institute for Health Research. UV=University of Valencia. HUB=Bellvitge University Hospital.

2.5 Supplementary Table 5. Diabetes pharmacotherapy pattern in each glycemic control subgroup of individuals with type 2 diabetes

Subgroup	Treatment combination at Baseline		Treatment combination after 1 year/ 3years	
	Code. Combination	N (%)	Code 1 year / Code 3 years	N (%)
S1. Good-Stable Glycemic Control Trajectory[a]	N	123 (100)		
	1. Metformin alone	59 (48.0)	1. / 1. (not changed)	43 (72.9)
			1. / 2.	3 (5.1)
			1. / 4.	1 (1.7)
			1. / 7.	1 (1.7)
			1. / NA.	1 (1.7)
			2. / 1.	1 (1.7)
			2. / 2.	4 (6.8)
			7. / 1.	1 (1.7)
			7. / 7.	3 (5.1)
			7. / NA.	1 (1.7)
	2. Metformin + Other*	26 (21.1)	2. / 2. (not changed)	24 (92.3)
			2. / NA.	1 (3.8)
			1. / 1.	1 (3.8)
	3. Metformin + Insulin	2 (1.6)	3. / 3. (not changed)	2 (100.0)
	4. Other treatments*	6 (4.9)	4. / 4. (not changed)	4 (66.7)
			4. / 2.	2 (33.3)
	5. Insulin alone	2 (1.6)	5. / 5. (not changed)	2 (100.0)
	6. Insulin + Other*	1 (0.8)	6. / 6. (not changed)	1 (100.0)
	7. No treatment	27 (22.0)	7. / 7. (not changed)	17 (63.0)
			7. / 1.	3 (11.1)
			1. / 1.	2 (7.4)
			1. / 7.	1 (3.7)
			7. / 4.	1 (3.7)
			4. / 7.	1 (3.7)
			1. / 2.	1 (3.7)
			7. / NA.	1 (3.7)
S2. Poor-Improved	N	17 (100)		

Glycemic Control Trajectory	2. Metformin + Other*	13 (76.5)	2. / 2. (not changed)	8 (61.5)
			2. / 3.	3 (23.1)
			3. / 3.	2 (15.4)
	1. Metformin alone	3 (17.6)	1. / 1. (not changed)	1 (33.3)
			1. / 7.	1 (33.3)
			1 / 2.	1 (33.3)
	3. Metformin + Insulin	1 (5.9)	3. / 3. (not changed)	1 (100)
S3. Poor-Worsen Glycemic Control Trajectory	N	8 (100)		
	3. Metformin + Insulin	4 (50.0)	3. / 3. (not changed)	2 (50.0)
			3. / 1.	1 (25.0)
			3. / 2.	1 (25.0)
	2. Metformin + Other*	3 (37.5)	2. / 2. (not changed)	1 (33.3)
			2. / 3.	1 (33.3)
			7. / 2.	1 (33.3)
	4. Other treatments*	1 (12.5)	4. / 4. (not changed)	1 (100)

N= number. NA= not available/missing.

[#]Other treatments included, in the overall sample: dipeptidyl peptidase 4 inhibitors (N=35), sulfonylureas (N=21), insulin secretagogues (N=11), SLGT2 inhibitors (N=6) and thiazolidinediones (N=1). Some participants took more than one of these treatments.

[a]N=87 participants from subgroup 1 were taking metformin at baseline, from those N=78 (89.7%) maintained the metformin treatment after 1 and 3 years of follow-up. From the 36 participants from subgroup 1 that were not taking metformin, N=26 (72.2%) continued without taking metformin throughout the 3 years of follow-up.

2.6 Supplementary Table 6. Comparability of individuals with type 2 diabetes from subgroup 1 treated and not treated with metformin before and after matching with inverse probability of treatment weights (IPTW)

		Individuals with type 2 diabetes from subgroup 1 [N=123]											
		Unmatched						Matched					
		No Metformin		Metformin					No Metformin	Metformin			
**Variable**	**Category**	**N**	**% (SD) / Mean (SD)**	**N**	**% (SD) / Mean (SD)**	**D#**	**P-value**	**% Rel Influence***	**% (SD) / Mean (SD)**	**% (SD) / Mean (SD)**	**D#**	**P-value**
N		36	100%	87	100%							
Study group	Intervention group	20	55.6 (0.5)	49	56.3 (0.5)	0.02	0.938	0.0	54.0 (0.5)	54.9 (0.5)	0.02	0.934
Gender	Women	18	50 (0.5)	45	51.7 (0.5)	0.03	0.863	0.0	51 (0.5)	52.6 (0.5)	0.031	0.883
Age	*Mean (SD)*	36	66.72 (5.16)	87	66.35 (4.39)	-0.08	0.884	0.2	66.51 (4.92)	66.31 (4.44)	-0.044	0.945
Education (years)	*Mean (SD)*	36	10.83 (4.02)	87	10.08 (3.95)	-0.19	0.491	1.2	10.94 (4)	10.06 (3.87)	-0.224	0.480
Current smoker	Yes	3	8.3 (0.28)	6	6.9 (0.25)	-0.06	0.782	0.0	7.2 (0.26)	6.5 (0.25)	-0.028	0.882
Intelligence Quotient	*Mean (SD)*	36	91.67 (44.38)	86	101.28 (38.72)	0.24	0.781	5.0	89.15 (48.78)	100.86 (38.75)	0.289	0.696
BMI (kg/m2)	*Mean (SD)*	36	32.23 (3.12)	87	33 (3.5)	0.23	0.083	14.6	32.75 (3.38)	32.81 (3.45)	0.018	0.737
Waist circumference	*Mean (SD)*	36	106.66 (8.6)	87	110.56 (10.45)	0.39	0.055	2.2	107.16 (8.55)	110.08 (10.3)	0.29	0.096
Total Cholesterol (mg/dL)	*Mean (SD)*	36	212.97 (40.26)	87	184.82 (31.99)	-0.77	**0.003**	12.6	203.92 (40.45)	187 (32.02)	-0.46	0.127
Triglycerides (mg/dL)	*Mean (SD)*	36	179.64 (80.87)	87	142.32 (79.47)	-0.46	0.061	14.7	170.36 (73.46)	144.47 (82.67)	-0.318	0.165
HDL-cholesterol (mg/dL)	*Mean (SD)*	36	48.03 (12.54)	87	49.86 (12.29)	0.15	0.553	3.9	47.53 (12.37)	50.13 (12.44)	0.211	0.529
LDL-cholesterol (mg/dL)	*Mean (SD)*	33	131.21 (37.56)	84	107.92 (29.64)	-0.69	**0.012**	10.9	123.54 (36.4)	109.75 (30.02)	-0.41	0.184
HbA1c (%)	*Mean (SD)*	36	6.56 (0.54)	78	6.61 (0.56)	0.10	0.897	31.2	6.59 (0.54)	6.61 (0.56)	0.028	0.995

BDI-II total score	*Mean (SD)*	36	9.25 (6.98)	87	9.15 (7.21)	-0.01	0.999	3.5	8.91 (6.72)	9.14 (7.11)	0.032	1
er-MedDiet adherence score	*Mean (SD)*	36	7.86 (2.43)	87	7.67 (2.65)	-0.08	0.995	0.0	7.66 (2.47)	7.73 (2.66)	0.028	1
Physical activity category	Sedentary	5	13.9 (0.35)	16	18.4 (0.39)	0.12	0.633	0.0	14.6 (0.35)	18.1 (0.39)	0.093	0.761
	Under-active	24	66.7 (0.47)	47	54 (0.5)	-0.26		0.0	64.1 (0.48)	54 (0.5)	-0.203	
	Moderately active	4	11.1 (0.31)	15	17.2 (0.38)	0.17		0.0	10.6 (0.31)	17.1 (0.38)	0.178	
	Active	3	8.3 (0.28)	9	10.3 (0.31)	0.07		0.0	10.7 (0.31)	10.8 (0.31)	0.004	
History of CVD (grouped)[1]		2	5.6 (0.23)	4	4.6 (0.21)	-0.04	0.824	0.0	4.4 (0.21)	4.3 (0.2)	-0.007	0.970
Angina pectoris		1		0								
Arrhythmia		2		4								
Other comorbidities (grouped)[2]		14	38.9 (0.49)	21	24.1 (0.43)	-0.33	0.103	0.0	36.7 (0.48)	25.2 (0.43)	-0.255	0.224
Claudication		0		2								
Emphysema		1		1								
Retinopathy		1		0								
Nephropathy		0		2								
Cholelithiasis		7		6								
Gout		6		3								
Cancer		1		9								
Depression		7	19.4 (0.4)	15	17.2 (0.38)	-0.06	0.773	0.0	18.9 (0.39)	16.7 (0.37)	-0.058	0.778
Sleep apnea		2	5.6 (0.23)	16	18.4 (0.39)	0.36	0.070	0.0	4.3 (0.2)	17.2 (0.38)	0.367	**0.036**
Insulin		3	8.3 (0.28)	2	2.3 (0.15)	-0.31	0.127	0.0	8.9 (0.29)	2 (0.14)	-0.349	0.076
Other treatments for diabetes (grouped)[3]		7	19.4 (0.4)	27	31 (0.46)	0.26	0.195	0.0	18.4 (0.39)	30.5 (0.46)	0.271	0.178
Dipeptidyl peptidase 4 inhibitors		5		22								

Sulfonylureas	2		4										
Insulin secretagogues	1		8										
SLGT2 inhibitors	0		2										
Medication for heart (grouped)[4]	7	19.4 (0.4)	16	18.4 (0.39)	-0.03	0.892	0.0	18.2 (0.39)	17.4 (0.38)	-0.021	0.917		
Medication for heart	2		5										
Treatment with aspirin/ Adiro®	5		12										
Medication for hypertension	28	77.8 (0.42)	70	80.5 (0.4)	0.07	0.738	0.0	80.7 (0.4)	80 (0.4)	-0.016	0.934		
Treatment for cholesterol	20	55.6 (0.5)	55	63.2 (0.48)	0.16	0.431	0.0	52.6 (0.5)	62.8 (0.48)	0.21	0.322		
Tranquilizers/ sedatives	11	30.6 (0.46)	24	27.6 (0.45)	-0.07	0.741	0.0	33.9 (0.47)	27.5 (0.45)	-0.143	0.508		

[1]Includes angina pectoris or arrhythmia

[2]Includes claudication, emphysema, retinopathy, nephropathy, cholelithiasis, gout and cancer

[3]Includes Dipeptidyl peptidase 4 inhibitors, Sulfonylureas, Insulin secretagogues and SLGT2 inhibitors

[4]Includes aspirin/ Adiro®, other

*Relative influence of each variable for estimating the probability of treatment assignment.

Standardized difference statistic.

N=number.

2.7 Supplementary Table 7. Differences in specific cognitive tests at each time point and in the mean change from baseline between individuals with type 2 diabetes from subgroup 1 (T2D-S1) treated with metformin and not treated with metformin (matched with inverse probability of treatment weights).

Variable	Time	T2D-S1 No-Metformin [N=36][1]		T2D-S1 Metformin [N=87][2]		Differences at each time point			Differences in the mean change (95%CI) from baseline		
		Missing [N (%)]	Mean (95%CI)	Missing [N (%)]	Mean (95%CI)	Cohen's D (95%CI)*	Effect Size[3]	P[4]	No-Metformin	Metformin	P[5]
RAVTL: immediate recall	Baseline	0 (0.0)	7 (5.8, 8.1)	1 (1.1)	7.2 (6.7, 7.7)	0.09 (-0.3, 0.48)	VS	0.764			
	1 year	6 (16.7)	7.1 (5.7, 8.4)	11 (12.6)	6.8 (6.3, 7.4)	-0.09 (-0.51, 0.34)	VS	0.548	0 (-1.1, 1)	-0.5 (-1.1, 0.1)	0.306
	3 years	6 (16.7)	8.4 (7.6, 9.3)	16 (18.4)	7.4 (6.8, 8)	-0.42 (-0.85, 0.03)	S	**0.019**	1.4 (0.4, 2.4)	-0.2 (-0.8, 0.5)	**0.022**
RAVTL: delayed recall	Baseline	0 (0.0)	6.9 (5.8, 7.9)	1 (1.1)	7.2 (6.6, 7.8)	0.11 (-0.28, 0.5)	VS	0.642			
	1 year	6 (16.7)	7.8 (6.4, 9.2)	11 (12.6)	6.9 (6.3, 7.5)	-0.31 (-0.74, 0.12)	S	0.143	0.8 (-0.2, 1.8)	-0.5 (-1.1, 0.2)	0.056
	3 years	6 (16.7)	8.3 (7.4, 9.2)	16 (18.4)	7.4 (6.6, 8.1)	-0.31 (-0.74, 0.13)	S	**0.051**	1.3 (0.2, 2.5)	-0.2 (-0.9, 0.5)	**0.034**
RCFT: immediate recall	Baseline	1 (2.8)	14.2 (11.6, 16.7)	3 (3.4)	17.0 (15.5, 18.5)	0.4 (-0.01, 0.8)	S	**0.050**			
	1 year	7 (19.4)	15.8 (12.4, 19.2)	12 (13.8)	18.7 (17, 20.3)	0.36 (-0.08, 0.79)	S	0.142	0.8 (-1.5, 3.2)	1.2 (-0.2, 2.5)	0.621
	3 years	6 (16.7)	17.1 (14, 20.1)	16 (18.4)	18.9 (17.5, 20.3)	0.26 (-0.17, 0.69)	S	0.349	1.8 (-0.4, 4)	1.3 (-0.1, 2.6)	0.399
RCFT: delayed recall	Baseline	1 (2.8)	13.3 (10.9, 15.8)	3 (3.4)	16.4 (15, 17.8)	0.45 (0.04, 0.85)	S	**0.032**			
	1 year	7 (19.4)	15.9 (12.8, 19)	13 (14.9)	17.6 (15.9, 19.4)	0.22 (-0.21, 0.66)	S	0.397	1.9 (-0.3, 4.1)	0.7 (-0.8, 2.2)	0.606
	3 years	6 (16.7)	16.5 (13.2, 19.7)	16 (18.4)	18.3 (16.9, 19.7)	0.27 (-0.17, 0.7)	S	0.424	2.2 (0.2, 4.2)	1.3 (0, 2.6)	0.244
RCFT: recognition	Baseline	0 (0.0)	18.4 (17.4, 19.4)	0 (0.0)	18.9 (18.3, 19.5)	0.17 (-0.22, 0.56)	S	0.548			
	1 year	6 (16.7)	19.5 (18.5, 20.5)	11 (12.6)	19.7 (19.2, 20.2)	0.09 (-0.33, 0.52)	VS	0.707	0.9 (-0.2, 2)	0.6 (0, 1.2)	0.718
	3 years	6 (16.7)	19.9 (19, 20.8)	16 (18.4)	19.8 (19.2, 20.3)	-0.05 (-0.47, 0.38)	VS	0.765	1.2 (0.2, 2.3)	0.9 (0.1, 1.6)	0.571
RCFT: Copy	Baseline	1 (2.8)	26.6 (23.5, 29.8)	1 (1.1)	30.6 (29.2, 32)	0.53 (0.11, 0.94)	M	**0.018**			
	1 year	7 (19.4)	26.7 (22.6, 30.7)	13 (14.9)	31.7 (30.7, 32.7)	0.72 (0.24, 1.18)	M	**0.017**	0.5 (-2, 3.1)	0.6 (-0.5, 1.6)	0.921

	3 years	6 (16.7)	28.4 (25.4, 31.5)	16 (18.4)	31.2 (30.1, 32.4)	0.45 (0.01, 0.89)	M	0.078	1.1 (-0.8, 3.1)	0.5 (-0.9, 1.8)	0.071
SMDT: direct	Baseline	7 (19.4)	34.6 (29.4, 39.8)	14 (16.1)	37.3 (34.3, 40.3)	0.2 (-0.24, 0.63)	S	0.486			
	1 year	7 (19.4)	32.3 (26, 38.6)	11 (12.6)	35.4 (32.6, 38.1)	0.22 (-0.21, 0.65)	S	0.464	-2 (-5, 1.1)	-0.1 (-2.2, 2)	0.536
	3 years	6 (16.7)	33.4 (27.8, 39.1)	17 (19.5)	35.1 (32.9, 37.3)	0.14 (-0.29, 0.57)	VS	0.709	-1.1 (-4.7, 2.4)	-1.5 (-4.4, 1.4)	0.937
Stroop: Interference	Baseline	0 (0.0)	1 (-2.4, 4.4)	0 (0.0)	2.6 (0, 5.1)	0.14 (-0.25, 0.53)	VS	0.362			
	1 year	6 (16.7)	2.2 (-1.3, 5.6)	11 (12.6)	1.4 (-0.7, 3.5)	-0.08 (-0.5, 0.34)	VS	0.699	0.8 (-3.2, 4.7)	-2.4 (-4.7, 0)	0.288
	3 years	6 (16.7)	0.9 (-4.5, 6.3)	16 (18.4)	-0.7 (-3.1, 1.7)	-0.14 (-0.56, 0.29)	VS	0.511	-1.1 (-6.5, 4.3)	-5.3 (-8.7, -2)	0.692
IGT: total	Baseline	1 (3.4)	-5.4 (-11.4, 0.5)	4 (6.6)	6.9 (1, 12.8)	0.6 (0.11, 1.07)	M	**0.002**			
	1 year	8 (27.6)	-4.6 (-12.8, 3.5)	12 (19.7)	8 (0.7, 15.3)	0.52 (-0.02, 1.05)	M	0.120	-2.2 (-12.1, 7.7)	2.8 (-4.5, 10)	0.652
	3 years	12 (41.4)	4.8 (-13, 22.6)	21 (34.4)	5.1 (-1.9, 12.2)	0.01 (-0.56, 0.58)	VS	0.855	15.9 (-3.8, 35.6)	-1.2 (-8.9, 6.5)	**0.015**
CPT: Omission errors	Baseline	2 (6.9)	11.3 (0.7, 21.9)	3 (4.9)	7 (3.4, 10.6)	-0.22 (-0.68, 0.24)	S	0.525			
	1 year	7 (24.1)	10.5 (-2.8, 23.8)	11 (18)	8.2 (3.7, 12.8)	-0.1 (-0.61, 0.4)	VS	0.805	1.2 (-1.4, 3.9)	2.4 (-1.2, 6)	0.807
	3 years	12 (41.4)	4.3 (1.1, 7.4)	27 (44.3)	7.7 (2.6, 12.8)	0.26 (-0.33, 0.85)	S	0.271	-7.1 (-20.6, 6.4)	1.7 (-3.1, 6.5)	0.923
CPT: Commission errors	Baseline	2 (6.9)	20 (12.8, 27.2)	3 (4.9)	21.4 (17.4, 25.3)	0.08 (-0.38, 0.54)	VS	0.840			
	1 year	7 (24.1)	20.5 (12, 29)	11 (18)	20.4 (15.4, 25.5)	-0.01 (-0.51, 0.5)	VS	0.966	-2.1 (-8.1, 3.9)	-0.9 (-3.7, 1.9)	0.998
	3 years	12 (41.4)	13.6 (8.5, 18.8)	27 (44.3)	14.8 (11.9, 17.6)	0.12 (-0.46, 0.7)	VS	0.611	-1.6 (-5, 1.8)	-2 (-4.5, 0.4)	0.935
CPT: HRT	Baseline	2 (6.9)	494.6 (448.3, 541)	4 (6.6)	463.8 (433.2, 494.5)	-0.26 (-0.72, 0.21)	S	0.318			
	1 year	7 (24.1)	471.4 (437.5, 505.3)	11 (18)	458.2 (431, 485.3)	-0.14 (-0.64, 0.36)	VS	0.461	11.7 (-15.3, 38.8)	-9.6 (-42, 22.8)	0.489
	3 years	12 (41.4)	452.5 (418.7, 486.2)	27 (44.3)	436.4 (397.3, 475.6)	-0.15 (-0.74, 0.43)	S	0.626	-7.2 (-54.5, 40.2)	-45.4 (-85.9, -5)	0.824
MMSE: total score	Baseline	0 (0.0)	28 (27.2, 28.8)	0 (0.0)	28.4 (28, 28.7)	0.18 (-0.22, 0.57)	S	0.450			

[1]N=29 and [2]N=61 when excluding participants from University of Valencia study site that did not receive all the tests from executive functions and global cognition (they did not perform the CPT and IGT tests).

[3]Effect Size: VS= very small (Cohen's d <0.2); S= small (Cohen's d (0.2-0.5)); M= medium (Cohen's d (0.5-0.8)); L= large (Cohen's d (0.8-1.2)); VL= very large (Cohen's d ≥ 1.2).

[4]ANOVA from multivariable-adjusted linear model.

[5]ANOVA from multivariable-adjusted linear mixed effects model.

Inverse probability of treatment weighting (IPTW) was applied to all analyses to weight each individual with his/her inverse probability of being treated with metformin, generating a pseudo-population with (almost) perfect covariate balance. All models were adjusted by diagnosis of sleep apnoea.

Reference group= No-Metformin

MMSE= Mini-Mental State Examination. RAVTL = Rey Auditory Verbal Learning Test. RCFT= Rey-Osterrieth Complex Figure Test. SDMT= Symbol Digit Modalities Test. CPT= Conner's Performance Task. IGT= Iowa Gambling Task.

2.8 Supplementary Table 8. Comparability of subjects with type 2 diabetes from subgroup 1 (T2D-S1) and subjects without type 2 diabetes (No-T2D)

Variable	Category	T2D-S1 N	T2D-S1 % (SD) / Mean (SD)	No-T2D N	No-T2D % (SD) / Mean (SD)	D#	P-value
N		123	100	339	100		
Study group	Intervention group	69	56.1 (0.5)	162	49.6 (0.5)	0.17	0.115
Gender	Women	63	51.2 (0.5)	173	50.0 (0.5)	0.00	0.972
Age	*Mean (SD)*	123	66.5 (4.6)	339	64.92 (4.7)	0.33	**0.015**
Education (years)	*Mean (SD)*	123	10.3 (4)	339	12.14 (5.7)	-0.34	**0.003**
Current smoker		9	7.3 (0.3)	47	13.9 (0.3)	-0.20	0.058
Intelligence Quotient	*Mean (SD)*	122	98.4 (40.5)	338	88.75 (39.5)	0.24	0.117
BMI (kg/m2)	*Mean (SD)*	123	32.8 (3.4)	339	32.37 (3.4)	0.12	0.475
Waist circumference	*Mean (SD)*	123	109.4 (10.1)	339	106.77 (9.9)	0.27	0.139
Total Cholesterol (mg/dL)	*Mean (SD)*	123	193.1 (36.8)	339	214.19 (39.3)	-0.53	**<0.001**
Triglycerides (mg/dL)	*Mean (SD)*	123	153.2 (81.4)	339	166.95 (100)	-0.14	0.564
HDL-cholesterol (mg/dL)	*Mean (SD)*	123	49.3 (12.3)	339	52.37 (12.6)	-0.24	**0.015**
LDL-cholesterol (mg/dL)	*Mean (SD)*	117	114.5 (33.6)	315	129.97 (33.3)	-0.46	**<0.001**
BDI-II total score	*Mean (SD)*	123	9.2 (7.1)	339	8.17 (6.7)	0.15	0.270
er-MedDiet adherence score	*Mean (SD)*	123	7.7 (2.6)	339	7.79 (2.5)	-0.03	0.945
Physical activity category	Sedentary	21	17.1 (0.4)	48	14.2 (0.3)	0.08	**0.030**
	Under-active	71	57.7 (0.5)	238	70.2 (0.5)	-0.27	
	Moderately active	19	15.4 (0.4)	25	7.4 (0.3)	0.28	
	Active	12	9.8 (0.3)	28	8.3 (0.3)	0.05	
History of CVD grouped[1]		6	4.9 (0.2)	22	6.5 (0.2)	-0.07	0.522
Angina pectoris		1		1			
Arrhythmia		6		21			
Other comorbidities grouped[2]		35	28.5 (0.5)	93	27.4 (0.4)	0.02	0.829
Claudication		2		0			
Emphysema		2		13			
Retinopathy		1		5			
Nephropathy		2		7			

Cholelithiasis	13		34			
Gout	9		31			
Cancer	10		18			
Depression	22	17.9 (0.4)	76	22.4 (0.4)	-0.11	0.293
Sleep apnea	18	14.6 (0.4)	46	13.6 (0.3)	0.03	0.770
Medication for heart grouped[4]	23	18.7 (0.4)	52	15.3 (0.4)	0.09	0.388
Medication for heart	7		21			
Treatment with aspirin/ Adiro®	17		33			
Medication for hypertension	98	79.7 (0.4)	251	74 (0.4)	0.13	0.214
Treatment for cholesterol	75	61 (0.5)	153	45.1 (0.5)	0.32	**0.003**
Tranquilizers/ sedatives	32	28.5 (0.5)	72	21.2 (0.4)	0.17	0.105

[1]Includes angina pectoris or arrhythmia

[2]Includes claudication, emphysema, retinopathy, nephropathy, cholelithiasis, gout and cancer

[3]Includes Dipeptidyl peptidase 4 inhibitors, Sulfonylureas, Insulin secretagogues and SLGT2 inhibitors

[4]Includes aspirin/ Adiro®, other

*Relative influence of each variable for estimating the probability of treatment assignment.

Standardized effect size, defined as the treatment group mean minus the control group divided by the pooled sample (treatment and control) standard deviation.

N=number.

2.9 Supplementary Table 9. Glycemic profile, MedDiet adherence, BMI and depressive symptomatology at baseline and after 1 and 3 years in the population with type 2 diabetes from subgroup 1 (T2D-S1), compared to the population without type 2 diabetes (No-T2D); and multivariable-adjusted differences

Variable	Time	Population	Missing N (%)	Mean (95%CI)	Between-group differences: Effect size difference[1] Cohen's D (95%CI)	Size	Model 1 (unadjusted) P-value	Model 2 (adjusted)[2] P-value
Glycosylated hemoglobin, HbA1c (%)	Baseline	No T2D	7 (2.1)	5.8 (5.7, 5.8)	Ref		**<0.001**	**<0.001**
		T2D-S1	9 (7.3)	6.6 (6.5, 6.7)	1.23 (0.99, 1.46)	VL		
	1 year	No T2D	22 (6.5)	5.7 (5.6, 5.7)	Ref		**<0.001**	**<0.001**
		T2D-S1	10 (8.1)	6.3 (6.2, 6.4)	0.96 (0.73, 1.19)	L		
	3 years	No T2D	46 (13.6)	5.8 (5.7, 5.9)	Ref		**<0.001**	**<0.001**
		T2D-S1	12 (9.8)	6.5 (6.4, 6.6)	0.94 (0.71, 1.18)	L		
Fasting plasma glucose (mg/dL)	Baseline	No T2D	0 (0.0)	103.3 (101.9, 104.7)	Ref		**<0.001**	**<0.001**
		T2D-S1	1 (0.8)	137.1 (132.5, 141.8)	8.31 (7.65, 8.97)	VL		
	1 year	No T2D	21 (6.2)	100.8 (99.3, 102.2)	Ref		**<0.001**	**<0.001**
		T2D-S1	10 (8.1)	126.9 (122.1, 131.8)	6.49 (5.93, 7.03)	VL		
	3 years	No T2D	44 (13)	103 (100.9, 105.1)	Ref		**<0.001**	**<0.001**
		T2D-S1	10 (8.1)	133.2 (128.1, 138.4)	6.61 (6.03, 7.18)	VL		
HOMA-IR index	Baseline	No T2D	7 (2.1)	5 (4.6, 5.3)	Ref		**<0.001**	**<0.001**
		T2D-S1	10 (8.1)	6.8 (5.9, 7.7)	0.96 (0.73, 1.18)	L		
	1 year	No T2D	24 (7.1)	4.1 (3.8, 4.4)			**<0.001**	**<0.001**
		T2D-S1	20 (16.3)	5.3 (4.6, 6)	0.71 (0.48, 0.94)	M		
er-MedDiet adherence	Baseline	No T2D	0 (0.0)	7.8 (7.5, 8.1)	Ref		0.789	0.684
		T2D-S1	0 (0.0)	7.7 (7.3, 8.2)	-0.04 (-0.25, 0.16)	VS		
	1 year	No T2D	12 (3.5)	11.7 (11.4, 12.1)	Ref		0.730	0.897
		T2D-S1	4 (3.3)	11.8 (11.3, 12.4)	0.06 (-0.15, 0.27)	VS		
	3 years	No T2D	33 (9.7)	11.6 (11.3, 12)	Ref		0.630	0.912
		T2D-S1	9 (7.3)	11.8 (11.3, 12.3)	0.09 (-0.12, 0.31)	VS		
BMI (kg/m^2)	Baseline	No T2D	0 (0.0)	32.4 (32, 32.7)	Ref		0.259	0.091
		T2D-S1	0 (0.0)	32.8 (32.2, 33.4)	0.22 (0.01, 0.43)	S		

	1 year	No T2D	2 (0.6)	31.0 (30.6, 31.4)	Ref		0.453	0.253
		T2D-S1	1 (0.8)	31.3 (30.6, 31.9)	0.15 (-0.06, 0.36)	S		
	3 years	No T2D	13 (3.8)	31.4 (31.0, 31.8)	Ref		0.844	0.910
		T2D-S1	4 (3.3)	31.3 (30.7, 32.0)	-0.04 (-0.25, 0.17)	VS		
Depressive symptomatology (BDI-II total score)	Baseline	No T2D	0 (0.0)	8.2 (7.5, 8.9)	Ref		0.156	0.228
		T2D-S1	0 (0.0)	9.2 (7.9, 10.4)	0.39 (0.18, 0.60)	S		
	1 year	No T2D	31 (9.1)	5.9 (5.3, 6.5)	Ref		**0.019**	**0.019**
		T2D-S1	10 (8.1)	7.4 (6.2, 8.6)	0.63 (0.41, 0.85)	M		
	3 years	No T2D	47 (13.9)	6.4 (5.6, 7.1)	Ref		0.853	0.741
		T2D-S1	12 (9.8)	6.2 (5, 7.5)	-0.05 (-0.27, 0.17)	VS		

[1]Effect Size: VS= very small (Cohen's d <0.2); S= small (Cohen's d (0.2-0.5)); M= medium (Cohen's d (0.5-0.8)); L= large (Cohen's d (0.8-1.2)); VL= very large (Cohen's d ≥ 1.2).

[2]ANOVA adjusted by age, education (years), current smoking status (smoker/non-smoker), intelligence quotient, waist, total cholesterol, HDL-cholesterol, LDL-cholesterol, physical activity category (sedentary, under-active, moderately active, active) and use of medications for cholesterol.

er-MedDiet= energy-restricted MedDiet adherence (from the adapted 17-item questionnaire). BDI=Beck's Depression Inventory -II. BMI= body mass index. HbA1c= glycosylated hemoglobin. 95%CI= 95% confidence interval.

2.10 Supplementary Table 10. Differences in MedDiet adherence and cognition at each time point and in the mean change from baseline between subjects without type 2 diabetes (No-T2D) and subjects with type 2 diabetes from subgroup 1 (T2D-S1).

Variable	Time	No-T2D [N=339][1]		T2D-S1 [N=123][2]		Differences at each time point			Differences in the mean change (95%CI) from baseline		
		Missing [N (%)]	Mean (95%CI)	Missing [N (%)]	Mean (95%CI)	Cohen's D (95%CI)	Size[3]	P[4]	No-T2D	T2D-S1	P[5]
er-MedDiet adherence score	Baseline	0 (0.0)	7.8 (7.5, 8.1)	0 (0.0)	7.7 (7.3, 8.2)	-0.04 (-0.25, 0.16)	VS	0.799			
	1 year	12 (3.5)	11.7 (11.4, 12.1)	4 (3.3)	11.8 (11.3, 12.4)	0.06 (-0.15, 0.27)	VS	0.885	4 (3.6, 4.3)	4.1 (3.5, 4.7)	0.476
	3 years	33 (9.7)	11.6 (11.3, 12)	9 (7.3)	11.8 (11.3, 12.3)	0.09 (-0.12, 0.31)	VS	0.748	3.8 (3.4, 4.1)	4.1 (3.6, 4.6)	0.144
Memory composite (z-score)	Baseline	5 (1.5)	-0.01 (-0.08, 0.06)	4 (3.3)	0.05 (-0.07, 0.17)	0.08 (-0.13, 0.29)	VS	0.864			
	1 year	50 (14.7)	0.19 (0.11, 0.28)	20 (16.3)	0.18 (0.06, 0.31)	-0.01 (-0.24, 0.21)	VS	0.954	0.19 (0.13, 0.25)	0.06 (-0.05, 0.17)	0.113
	3 years	83 (24.5)	0.56 (0.48, 0.65)	22 (17.9)	0.34 (0.21, 0.47)	-0.27 (-0.50, -0.04)	S	**0.034**	0.55 (0.47, 0.63)	0.10 (-0.04, 0.25)	**<0.001**
Executive functions composite (z-score)[1,2]	Baseline	41 (13.4)	-0.06 (-0.12, -0.01)	31 (34.4)	0.02 (-0.11, 0.16)	0.13 (-0.15, 0.41)	VS	0.778			
	1 year	74 (24.3)	0.01 (-0.05, 0.07)	24 (26.7)	0.05 (-0.07, 0.17)	0.07 (-0.21, 0.34)	VS	0.960	0.03 (-0.01, 0.08)	0.03 (-0.06, 0.13)	0.878
	3 years	154 (50.5)	0.08 (0, 0.16)	42 (46.7)	0.18 (0.05, 0.32)	0.15 (-0.18, 0.47)	VS	0.404	0.08 (0, 0.16)	0.02 (-0.11, 0.14)	0.747
Global cognition composite (z-score)[1,2]	Baseline	43 (14.1)	-0.04 (-0.1, 0.02)	32 (35.6)	0.05 (-0.07, 0.17)	0.13 (-0.16, 0.41)	VS	0.404			
	1 year	78 (25.6)	0.10 (0.03, 0.16)	25 (27.8)	0.15 (0.03, 0.27)	0.07 (-0.20, 0.35)	VS	0.740	0.11 (0.07, 0.16)	0.13 (0.05, 0.22)	0.547
	3 years	155 (50.8)	0.30 (0.23, 0.37)	42 (46.7)	0.25 (0.12, 0.39)	-0.07 (-0.39, 0.26)	VS	0.260	0.25 (0.18, 0.32)	-0.03 (-0.12, 0.07)	0.075
RAVTL: immediate recall	Baseline	0 (0.0)	7.6 (7.3, 7.9)	1 (0.8)	7.3 (6.8, 7.7)	-0.17 (-0.38, 0.03)	S	0.202			
	1 year	45 (13.3)	8 (7.6, 8.3)	17 (13.8)	6.9 (6.4, 7.5)	-0.62 (-0.84, -0.39)	M	0.059	0.5 (0.2, 0.8)	-0.4 (-1, 0.1)	**0.017**
	3 years	81 (23.9)	9.4 (9, 9.8)	22 (17.9)	7.7 (7.2, 8.2)	-0.96 (-1.2, -0.71)	L	**0.001**	1.8 (1.4, 2.2)	-0.3 (-0.9, 0.4)	**<0.001**
RAVTL: delayed recall	Baseline	0 (0.0)	7.4 (7.1, 7.7)	1 (0.8)	7.2 (6.7, 7.7)	-0.13 (-0.33, 0.08)	VS	0.77			
	1 year	45 (13.3)	7.7 (7.3, 8)	17 (13.8)	7.2 (6.6, 7.7)	-0.3 (-0.53, -0.08)	S	0.664	0.3 (0, 0.6)	-0.1 (-0.7, 0.5)	0.201

	3 years	81 (23.9)	9.4 (9, 9.8)	22 (17.9)	7.7 (7.2, 8.3)	-0.92 (-1.16, -0.68)	L	**0.005**	2 (1.6, 2.4)	-0.2 (-0.9, 0.5)	**<0.001**
RCFT: immediate recall	Baseline	4 (1.2)	13.9 (13.1, 14.6)	4 (3.3)	16.4 (15.1, 17.7)	0.95 (0.73, 1.17)	L	**0.033**			
	1 year	47 (13.9)	15.4 (14.6, 16.2)	19 (15.4)	18.1 (16.6, 19.5)	0.99 (0.75, 1.22)	L	**0.019**	1.1 (0.5, 1.8)	1 (-0.1, 2.2)	0.669
	3 years	81 (23.9)	17.1 (16.3, 17.9)	22 (17.9)	18.8 (17.5, 20)	0.66 (0.42, 0.9)	M	0.093	2.9 (2.2, 3.6)	1.3 (0, 2.7)	0.131
RCFT: delayed recall	Baseline	5 (1.5)	13.6 (12.9, 14.3)	4 (3.3)	15.7 (14.5, 16.9)	0.8 (0.59, 1.02)	L	0.086			
	1 year	50 (14.7)	15 (14.2, 15.8)	20 (16.3)	17.3 (15.9, 18.7)	0.89 (0.66, 1.13)	L	**0.049**	1.1 (0.5, 1.7)	1 (-0.1, 2.2)	0.723
	3 years	83 (24.5)	17.2 (16.4, 18)	22 (17.9)	18.2 (16.9, 19.5)	0.38 (0.15, 0.62)	S	0.305	3.4 (2.7, 4.2)	1.3 (0, 2.7)	0.092
RCFT: recognition	Baseline	0 (0.0)	19.2 (19, 19.5)	0 (0.0)	18.8 (18.3, 19.3)	-0.28 (-0.49, -0.07)	S	0.154			
	1 year	45 (13.3)	20.2 (19.9, 20.4)	17 (13.8)	19.7 (19.3, 20.2)	-0.3 (-0.53, -0.08)	S	0.111	0.9 (0.6, 1.2)	0.7 (0.1, 1.2)	0.467
	3 years	81 (23.9)	20.5 (20.2, 20.7)	22 (17.9)	19.9 (19.4, 20.3)	-0.42 (-0.65, -0.18)	S	**0.037**	1.1 (0.8, 1.4)	0.9 (0.1, 1.6)	0.590
RCFT: Copy	Baseline	4 (1.2)	26.8 (26, 27.6)	2 (1.6)	29.7 (28.4, 30.9)	1.04 (0.82, 1.27)	L	**0.039**			
	1 year	45 (13.3)	27.4 (26.6, 28.3)	20 (16.3)	30.5 (29.2, 31.8)	1.13 (0.89, 1.37)	L	**0.015**	0.3 (-0.3, 0.9)	0.6 (-0.4, 1.5)	0.728
	3 years	81 (23.9)	29.5 (28.9, 30.2)	22 (17.9)	30.7 (29.6, 31.8)	0.5 (0.26, 0.73)	M	0.238	2.8 (2, 3.6)	0.3 (-0.9, 1.5)	**0.009**
SMDT: direct	Baseline	25 (7.4)	36.8 (35.4, 38.2)	21 (17.1)	36.7 (34.1, 39.3)	-0.03 (-0.25, 0.2)	VS	0.07			
	1 year	51 (15)	36 (34.6, 37.4)	18 (14.6)	34.8 (32.3, 37.2)	-0.35 (-0.58, -0.13)	S	0.636	-0.9 (-1.9, 0.2)	-0.7 (-2.5, 1)	0.416
	3 years	81 (23.9)	34.2 (32.8, 35.7)	23 (18.7)	34.7 (32.5, 36.9)	0.13 (-0.1, 0.37)	VS	0.25	-2.5 (-3.5, -1.4)	-1.6 (-4.4, 1.3)	0.746
Stroop: Interference	Baseline	0 (0.0)	-1.3 (-2.3, -0.3)	0 (0.0)	1.9 (-0.1, 3.9)	1.02 (0.8, 1.24)	L	0.07			
	1 year	45 (13.3)	-1.1 (-2, -0.1)	17 (13.8)	1.7 (-0.1, 3.4)	0.95 (0.71, 1.18)	L	**0.003**	-0.1 (-1.1, 0.9)	-1.2 (-3.2, 0.7)	0.655
	3 years	81 (23.9)	-1.2 (-2.1, -0.2)	22 (17.9)	-0.8 (-2.7, 1.2)	0.14 (-0.1, 0.37)	VS	0.84	-0.5 (-1.7, 0.8)	-5.1 (-8.2, -1.9)	**0.005**
IGT: total	Baseline	13 (4.3)	1.7 (-0.8, 4.2)	5 (5.6)	2.2 (-2.6, 6.9)	0.11 (-0.13, 0.35)	VS	0.549			
	1 year	62 (20.3)	3 (0.1, 5.9)	20 (22.2)	3.8 (-2.2, 9.8)	0.17 (-0.1, 0.43)	S	0.899	0.9 (-3, 4.7)	1.8 (-4.6, 8.3)	0.922
	3 years	142 (46.6)	7.2 (3.7, 10.7)	33 (36.7)	5.4 (-1.6, 12.3)	-0.38 (-0.68, -0.07)	S	0.372	4.5 (-0.2, 9.2)	-2.3 (-10.1, 5.6)	0.660
CPT: Omission errors	Baseline	5 (1.6)	7.3 (5.6, 9)	5 (5.6)	7.6 (4.2, 10.9)	0.08 (-0.16, 0.32)	VS	0.782			
	1 year	57 (18.7)	5.7 (4.4, 7.1)	18 (20)	8 (4.1, 11.9)	0.66 (0.39, 0.93)	M	0.226	-1.6 (-3.3, 0.1)	2 (-0.8, 4.8)	0.094

	3 years	145 (47.5)	7.7 (4.2, 11.2)	39 (43.3)	6.2 (3.1, 9.4)	-0.32 (-0.64, -0.01)	S	0.266	1.2 (-2.2, 4.7)	1.2 (-2.8, 5.1)	0.631
CPT: Commission errors	Baseline	5 (1.6)	22.9 (21.1, 24.7)	5 (5.6)	21 (17.6, 24.5)	-0.45 (-0.7, -0.21)	M	0.937			
	1 year	57 (18.7)	20.4 (18.5, 22.3)	18 (20)	19.9 (16.1, 23.8)	-0.12 (-0.38, 0.14)	VS	0.887	-2 (-3.5, -0.6)	-1.5 (-3.7, 0.8)	0.938
	3 years	145 (47.5)	17.3 (15.3, 19.4)	39 (43.3)	14.7 (12, 17.4)	-0.76 (-1.08, -0.43)	L	0.331	-3.4 (-5.3, -1.5)	-1.9 (-4.5, 0.6)	0.694
CPT: HRT	Baseline	6 (2)	457.9 (449.2, 466.6)	6 (6.7)	473.6 (451.5, 495.8)	1.74 (1.46, 2.02)	VL	**0.042**			
	1 year	59 (19.3)	459 (450.1, 468)	18 (20)	461.9 (440.6, 483.3)	0.33 (0.07, 0.6)	S	0.626	1.8 (-7.8, 11.4)	-5.5 (-27.3, 16.3)	0.094
	3 years	145 (47.5)	454.5 (440.5, 468.5)	39 (43.3)	437.1 (409.2, 465.1)	-1.81 (-2.18, -1.43)	VL	0.854	-0.5 (-12.9, 12)	-48.5 (-87.6, -9.3)	**0.016**
MMSE: total score	Baseline	5 (1.5)	28.7 (28.5, 28.9)	0 (0.0)	28.3 (28, 28.7)	-0.27 (-0.48, -0.06)	S	0.255			

[1]N=305 and [2]N=90 when excluding participants from University of Valencia study site that did not perform the CPT and IGT.

[3]Effect Size: VS= very small (Cohen's d <0.2); S= small (Cohen's d (0.2-0.5)); M= medium (Cohen's d (0.5-0.8)); L= large (Cohen's d (0.8-1.2)); VL= very large (Cohen's d ≥ 1.2).

[4]ANOVA from multivariable-adjusted linear model

[5]ANOVA from multivariable-adjusted linear model mixed effects model.

All models were adjusted by the following baseline characteristics: age, education (years), current smoking status (smoker/non-smoker), intelligence quotient, waist, total cholesterol, HDL-cholesterol, LDL-cholesterol, physical activity category (sedentary, under-active, moderately active, active) and use of medications for cholesterol.

**Reference group= population without type 2 diabetes.*

MMSE= Mini-Mental State Examination. RAVTL = Rey Auditory Verbal Learning Test. RCFT= Rey-Osterrieth Complex Figure Test. SDMT= Symbol Digit Modalities Test. CPT= Conner's Performance Task. IGT= Iowa Gambling Task.

2.11 Supplementary Table 11. Comparability between the population without type 2 diabetes (No-T2D) and the population with type 2 diabetes from subgroup 1 (T2D-S1) treated with Metformin before and after matching with inverse probability of treatment weights (IPTW)

		Unmatched						Matched				
		No-T2D		T2D-S1 Metformin		D#	P-value	% Rel	No-T2D	T2D-S1 Metformin	D#	P-value
Variable	Category	N	% (SD) / Mean (SD)	N	% (SD) / Mean (SD)			Influence*	% (SD) / Mean (SD)	% (SD) / Mean (SD)		
N		339	100	87	100							
Study group	Intervention group	162	47.8 (0.5)	49	56.3 (0.5)	0.171	0.157	0.0	48.4 (0.5)	58.8 (0.5)	0.21	0.125
Gender	Men	166	49 (0.5)	42	48.3 (0.5)	-0.014	0.908	1.0	48.9 (0.5)	39 (0.5)	-0.20	0.134
Age	*Mean (SD)*	339	64.92 (4.7)	87	66.35 (4.4)	0.307	0.058	5.4	65.06 (4.7)	66.04 (4.3)	0.21	0.304
Education (years)	*Mean (SD)*	339	12.14 (5.7)	87	10.08 (3.9)	-0.376	**0.002**	10.5	11.93 (5.6)	9.9 (4.1)	-0.37	0.086
Current smoker	Yes	47	13.9 (0.3)	6	6.9 (0.3)	-0.211	0.08	1.1	13.4 (0.3)	5 (0.2)	-0.25	**0.017**
Intelligence Quotient	*Mean (SD)*	338	88.75 (39.5)	86	101.28 (38.7)	0.316	0.08	14.3	89.54 (39.2)	94.16 (37.6)	0.12	0.945
BMI (kg/m2)	*Mean (SD)*	339	32.37 (3.4)	87	33 (3.5)	0.184	0.236	7.5	32.41 (3.4)	32.97 (3.3)	0.16	0.424
Waist circumference	*Mean (SD)*	339	106.77 (9.9)	87	110.56 (10.4)	0.375	**0.034**	6.5	107 (9.9)	109.31 (9.3)	0.23	0.174
Total Cholesterol (mg/dL)	*Mean (SD)*	339	214.19 (39.3)	87	184.82 (32)	-0.741	**<0.001**	25.1	211.4 (39.6)	195.34 (32.8)	-0.41	0.06
Triglycerides (mg/dL)	*Mean (SD)*	339	166.95 (100)	87	142.32 (79.5)	-0.255	0.122	5.2	164.38 (97.6)	142.11 (74.1)	-0.23	0.184
HDL-cholesterol (mg/dL)	*Mean (SD)*	339	52.37 (12.6)	87	49.86 (12.3)	-0.2	0.122	3.4	52.21 (12.5)	52.18 (12.5)	0.00	0.995
LDL-cholesterol (mg/dL)	*Mean (SD)*	315	129.97 (33.3)	84	107.92 (29.6)	-0.653	**<0.001**	8.3	127.63 (33.5)	116.27 (30.5)	-0.34	0.101
BDI-II total score	*Mean (SD)*	339	8.17 (6.7)	87	9.15 (7.2)	0.145	0.512	4.5	8.28 (6.6)	8.74 (6.9)	0.07	0.992
er-MedDiet adherence score	*Mean (SD)*	339	7.79 (2.5)	87	7.67 (2.7)	-0.051	0.987	0.2	7.8 (2.4)	7.82 (2.6)	0.01	0.999
Physical activity category	Sedentary	48	14.2 (0.3)	16	18.4 (0.4)	0.118	**0.012**	6.5	14.1 (0.3)	14.9 (0.4)	0.02	0.596

Under-active	238	70.2 (0.5)	47	54 (0.5)	-0.344		0.0	69.4 (0.5)	62.6 (0.5)	-0.14	
Moderately active	25	7.4 (0.3)	15	17.2 (0.4)	0.338		0.0	7.9 (0.3)	11.7 (0.3)	0.13	
Active	28	8.3 (0.3)	9	10.3 (0.3)	0.074		0.0	8.7 (0.3)	10.8 (0.3)	0.08	
History of CVD grouped[1]	22	6.5 (0.2)	4	4.6 (0.2)	-0.079	0.512	0.0	6.5 (0.2)	3.3 (0.2)	-0.13	0.216
Angina pectoris	1		0								
Arrhythmia	21		4								
Other comorbidities grouped[2]	93	27.4 (0.4)	21	24.1 (0.4)	-0.074	0.536	0.0	27.1 (0.4)	21.4 (0.4)	-0.13	0.305
Claudication	0		2								
Emphysema	13		1								
Retinopathy	5		0								
Nephropathy	7		2								
Cholelithiasis	34		6								
Gout	31		3								
Cancer	18		9								
Depression	76	22.4 (0.4)	15	17.2 (0.4)	-0.126	0.294	0.0	21.9 (0.4)	22.3 (0.4)	0.01	0.949
Sleep apnea	46	13.6 (0.3)	16	18.4 (0.4)	0.137	0.256	0.1	13.9 (0.3)	13.8 (0.3)	0.00	0.982
Medication for heart grouped[3]	52	15.3 (0.4)	16	18.4 (0.4)	0.083	0.489	0.1	16.1 (0.4)	15.8 (0.4)	-0.01	0.937
Medication for heart	21		5								
Treatment with aspirin/Adiro®	33		12								
Medication for hypertension	251	74 (0.4)	70	80.5 (0.4)	0.149	0.216	0.0	74.8 (0.4)	77.2 (0.4)	0.05	0.714
Treatment for cholesterol	153	45.1 (0.5)	55	63.2 (0.5)	0.362	**0.003**	0.0	46.7 (0.5)	62.6 (0.5)	0.32	**0.024**
Tranquilizers/ sedatives	72	21.2 (0.4)	24	27.6 (0.4)	0.152	0.207	0.2	21.9 (0.4)	27.9 (0.4)	0.14	0.314

[1]Includes angina pectoris or arrhythmia

[2]Includes claudication, emphysema, retinopathy, nephropathy, cholelithiasis, gout and cancer

[3]Includes aspirin/ Adiro®, other

*Relative influence of each variable for estimating the probability of treatment assignment.

Standardized difference statistic.

N= number.

2.12 Supplementary Table 12. Differences in MedDiet adherence and cognition at each time point and in the mean change from baseline between subjects without type 2 diabetes (No-T2D) and subjects with type 2 diabetes from subgroup 1 (T2D-S1, with ‘Good-Stable’ glycemic control trajectory) treated with metformin (matched analyses with inverse probability of treatment weights)

Variable	Time	No-T2D [N=339][1]		T2D-S1 Metformin [N=87][2]		Differences at each time point			Differences in the mean change (95%CI) from baseline		
		Missing [N (%)]	Mean (95%CI)	Missing [N (%)]	Mean (95%CI)	Cohen's D (95%CI)*	Effect Size[3]	P[4]	No-T2D	T2D-S1 Metformin	P[5]
er-MedDiet adherence score	Baseline	0 (0.0)	7.8 (7.5, 8.1)	0 (0.0)	7.8 (7.2, 8.4)	0.01 (-0.23, 0.24)	VS	0.942			
	1 year	12 (3.5)	11.7 (11.4, 12)	3 (3.4)	11.9 (11.1, 12.7)	0.06 (-0.18, 0.3)	VS	0.817	3.9 (3.5, 4.3)	4.1 (3.1, 5.1)	0.921
	3 years	33 (9.7)	11.6 (11.3, 12)	8 (9.2)	11.6 (11, 12.2)	-0.01 (-0.26, 0.24)	VS	0.293	3.8 (3.4, 4.1)	4.0 (3.3, 4.7)	0.765
Memory composite (z-score)	Baseline	5 (1.5)	-0.01 (-0.08, 0.07)	3 (3.4)	0.09 (-0.05, 0.22)	0.14 (-0.1, 0.38)	VS	0.170			
	1 year	50 (14.7)	0.19 (0.11, 0.27)	13 (14.9)	0.13 (-0.04, 0.3)	-0.09 (-0.35, 0.17)	VS	0.847	0.18 (0.12, 0.24)	-0.02 (-0.16, 0.11)	**0.040**
	3 years	83 (24.5)	0.55 (0.46, 0.64)	16 (18.4)	0.29 (0.14, 0.45)	-0.37 (-0.63, -0.1)	S	0.091	0.53 (0.44, 0.61)	0.14 (-0.02, 0.31)	**<0.001**
Executive functions composite (z-score)[1,2]	Baseline	41 (13.4)	-0.06 (-0.12, -0.01)	21 (34.4)	0.01 (-0.23, 0.24)	0.14 (-0.19, 0.48)	VS	0.399			
	1 year	74 (24.3)	0.01 (-0.05, 0.06)	14 (23.0)	0.05 (-0.09, 0.2)	0.11 (-0.21, 0.42)	VS	0.195	0.03 (-0.01, 0.07)	-0.02 (-0.16, 0.13)	0.736
	3 years	154 (50.5)	0.08 (0.00, 0.16)	28 (45.9)	0.11 (-0.07, 0.29)	0.06 (-0.32, 0.44)	VS	0.173	0.08 (0, 0.16)	0.02 (-0.08, 0.13)	0.454
Global cognition composite (z-score)[1,2]	Baseline	43 (14.1)	-0.04 (-0.09, 0.02)	22 (36.1)	0.04 (-0.15, 0.23)	0.16 (-0.17, 0.5)	S	0.362			
	1 year	78 (25.6)	0.10 (0.03, 0.16)	15 (24.6)	0.11 (-0.04, 0.27)	0.04 (-0.28, 0.35)	VS	0.490	0.11 (0.07, 0.15)	0.1 (-0.03, 0.22)	0.826
	3 years	155 (50.8)	0.30 (0.23, 0.37)	28 (45.9)	0.18 (-0.01, 0.36)	-0.26 (-0.64, 0.12)	S	0.553	0.25 (0.18, 0.32)	-0.01 (-0.12, 0.11)	**0.003**
RAVTL: immediate recall	Baseline	0 (0.0)	7.6 (7.3, 7.9)	1 (1.1)	7.3 (6.8, 7.9)	-0.09 (-0.32, 0.15)	VS	0.451			
	1 year	45 (13.3)	7.9 (7.6, 8.3)	11 (12.6)	6.9 (6.3, 7.6)	-0.34 (-0.6, -0.09)	S	0.067	0.4 (0.1, 0.7)	-0.4 (-1, 0.1)	**0.006**
	3 years	81 (23.9)	9.3 (8.9, 9.7)	16 (18.4)	7.6 (6.9, 8.3)	-0.52 (-0.79, -0.25)	M	**0.004**	1.7 (1.3, 2.1)	0.1 (-0.7, 0.8)	**<0.001**
RAVTL: delayed recall	Baseline	0 (0.0)	7.4 (7, 7.7)	1 (1.1)	7.5 (6.9, 8.1)	0.06 (-0.18, 0.29)	VS	0.481			

	1 year	45 (13.3)	7.7 (7.3, 8)	11 (12.6)	6.8 (6.1, 7.4)	-0.28 (-0.54, -0.03)	S	0.111	0.3 (0, 0.6)	-0.8 (-1.5, -0.1)	**0.054**
	3 years	81 (23.9)	9.3 (8.9, 9.7)	16 (18.4)	7.7 (6.8, 8.5)	-0.47 (-0.74, -0.21)	M	**0.013**	2 (1.6, 2.4)	-0.1 (-0.9, 0.6)	**<0.001**
RCFT: immediate recall	Baseline	4 (1.2)	14 (13.3, 14.8)	3 (3.4)	16.3 (14.6, 17.9)	0.32 (0.08, 0.56)	S	**0.021**			
	1 year	47 (13.9)	15.5 (14.7, 16.3)	12 (13.8)	17.3 (15.6, 19.1)	0.25 (-0.01, 0.5)	S	0.081	1.1 (0.5, 1.7)	0.4 (-1, 1.8)	0.533
	3 years	81 (23.9)	17.1 (16.2, 17.9)	16 (18.4)	18.1 (16.6, 19.7)	0.16 (-0.1, 0.42)	S	0.114	2.7 (1.9, 3.5)	1.5 (0, 3)	0.156
RCFT: delayed recall	Baseline	5 (1.5)	13.7 (13, 14.4)	3 (3.4)	15.6 (14.1, 17.1)	0.27 (0.03, 0.51)	S	**0.037**			
	1 year	50 (14.7)	15.1 (14.3, 15.9)	13 (14.9)	16.6 (14.8, 18.4)	0.22 (-0.04, 0.48)	S	0.129	1.1 (0.5, 1.7)	0.5 (-0.9, 1.9)	0.977
	3 years	83 (24.5)	17.1 (16.3, 18)	16 (18.4)	17.5 (16.1, 18.9)	0.06 (-0.21, 0.32)	VS	0.275	3.2 (2.4, 4)	1.5 (0.3, 2.7)	**0.050**
RCFT: recognition	Baseline	0 (0.0)	19.2 (19, 19.5)	0 (0.0)	19 (18.4, 19.5)	-0.12 (-0.36, 0.12)	VS	0.735			
	1 year	45 (13.3)	20.1 (19.9, 20.4)	11 (12.6)	19.8 (19.3, 20.4)	-0.15 (-0.41, 0.1)	S	0.475	0.8 (0.5, 1.1)	0.6 (0, 1.2)	0.664
	3 years	81 (23.9)	20.5 (20.2, 20.7)	16 (18.4)	19.9 (19.3, 20.5)	-0.27 (-0.53, -0.01)	S	0.199	1.1 (0.8, 1.4)	0.8 (0.1, 1.6)	0.633
RCFT: Copy	Baseline	4 (1.2)	27 (26.2, 27.8)	1 (1.1)	30.2 (28.8, 31.6)	0.43 (0.2, 0.67)	S	**0.002**			
	1 year	45 (13.3)	27.6 (26.7, 28.5)	13 (14.9)	30.7 (29.3, 32.2)	0.42 (0.17, 0.68)	S	**<0.001**	0.3 (-0.3, 0.9)	0.3 (-1.4, 1.9)	0.642
	3 years	81 (23.9)	29.6 (28.9, 30.2)	16 (18.4)	30.9 (29.7, 32.2)	0.25 (-0.01, 0.51)	S	0.074	2.6 (1.8, 3.4)	0.5 (-0.6, 1.5)	**0.007**
SMDT: direct	Baseline	25 (7.4)	36.5 (35.1, 37.9)	14 (16.1)	36.9 (33.1, 40.6)	0.03 (-0.23, 0.28)	VS	0.091			
	1 year	51 (15)	35.8 (34.4, 37.2)	11 (12.6)	34.8 (31.8, 37.8)	-0.08 (-0.33, 0.17)	VS	0.469	-0.8 (-1.8, 0.3)	-0.2 (-2.8, 2.3)	0.595
	3 years	81 (23.9)	34.1 (32.6, 35.5)	17 (19.5)	35.2 (33, 37.5)	0.1 (-0.16, 0.37)	VS	**0.009**	-2.4 (-3.4, -1.4)	-1.6 (-5.1, 1.9)	0.804
Stroop: Interference	Baseline	0 (0.0)	-1.1 (-2.1, -0.1)	0 (0.0)	1.4 (-1.4, 4.2)	0.24 (0, 0.47)	S	0.226			
	1 year	45 (13.3)	-1 (-2, -0.1)	11 (12.6)	1.3 (-1, 3.6)	0.27 (0.02, 0.52)	S	0.063	-0.2 (-1.2, 0.8)	-1.2 (-3.9, 1.5)	0.201
	3 years	81 (23.9)	-1.2 (-2.1, -0.2)	16 (18.4)	-0.1 (-2.6, 2.5)	0.13 (-0.13, 0.39)	VS	0.541	-0.7 (-2.1, 0.6)	-3.9 (-7.4, -0.3)	**0.002**
IGT: total	Baseline	13 (4.3)	1.7 (-0.8, 4.2)	4 (6.6)	6.5 (1, 12.1)	0.22 (-0.06, 0.51)	S	0.187			
	1 year	62 (20.3)	3 (0.2, 5.8)	12 (19.7)	8.8 (0.8, 16.8)	0.25 (-0.06, 0.55)	S	0.110	0.7 (-3.1, 4.5)	3.8 (-4.2, 11.7)	0.859
	3 years	142 (46.6)	7.4 (3.9, 10.8)	21 (34.4)	4.6 (-2.3, 11.5)	-0.12 (-0.47, 0.22)	VS	0.715	4.5 (-0.1, 9.2)	-2 (-9, 5)	0.194
CPT: Omission errors	Baseline	5 (1.6)	7.3 (5.6, 8.9)	3 (4.9)	12.2 (1.2, 23.3)	0.23 (-0.05, 0.51)	S	0.393			

	1 year	57 (18.7)	5.9 (4.5, 7.3)	11 (18)	10.1 (3.8, 16.5)	0.31 (0, 0.61)	S	0.341	-1.5 (-3.2, 0.3)	3.5 (-1.6, 8.5)	0.152
	3 years	145 (47.5)	7.8 (4.3, 11.3)	27 (44.3)	8.9 (1.8, 16)	0.05 (-0.32, 0.42)	VS	0.874	1.3 (-2.2, 4.7)	2.5 (-4.2, 9.3)	0.909
CPT: Commission errors	Baseline	5 (1.6)	22.6 (20.8, 24.4)	3 (4.9)	20 (15.8, 24.2)	-0.16 (-0.44, 0.12)	S	0.559			
	1 year	57 (18.7)	20.2 (18.3, 22.1)	11 (18)	20.4 (15.1, 25.8)	0.01 (-0.29, 0.32)	VS	0.963	-2 (-3.4, -0.6)	0.1 (-3.8, 4)	0.714
	3 years	145 (47.5)	17.1 (15, 19.2)	27 (44.3)	13.5 (10.6, 16.4)	-0.28 (-0.65, 0.09)	S	0.325	-3.3 (-5.2, -1.4)	-2 (-4.4, 0.4)	0.454
CPT: HRT	Baseline	6 (2)	458.3 (449.6, 466.9)	4 (6.6)	479.4 (440.1, 518.6)	0.23 (-0.06, 0.51)	S	0.179			
	1 year	59 (19.3)	459.1 (450, 468.2)	11 (18)	450.5 (402.1, 498.9)	-0.09 (-0.39, 0.22)	VS	0.670	1.4 (-8.4, 11.1)	-28.6 (-84.5, 27.2)	0.418
	3 years	145 (47.5)	454.4 (440.2, 468.6)	27 (44.3)	451.6 (403.8, 499.4)	-0.03 (-0.4, 0.34)	VS	0.362	-1.3 (-14, 11.5)	-34.1 (-81.1, 12.9)	**0.016**
MMSE: total score	Baseline	5 (1.5)	28.7 (28.5, 28.8)	0 (0.0)	28.4 (27.9, 28.9)	-0.16 (-0.4, 0.08)	S	0.739			

[1]N=305 and [2]N=61 when excluding participants from University of Valencia study site that did not perform the CPT and IGT.

[3]Effect Size: VS= very small (Cohen's d <0.2); S= small (Cohen's d (0.2-0.5)); M= medium (Cohen's d (0.5-0.8)); L= large (Cohen's d (0.8-1.2)); VL= very large (Cohen's d ≥ 1.2).

[4]ANOVA from multivariable-adjusted linear model

[5]ANOVA from multivariable-adjusted linear mixed effects model.

All models were adjusted by years of education, smoking status, total cholesterol, LDL-cholesterol, and use of treatment for high cholesterol.

Inverse probability of treatment weighting (IPTW) was applied to all analyses to weight each individual with his/her inverse probability of being treated with metformin, generating a pseudo-population with (almost) perfect

covariate balance.

Reference group= population without type 2 diabetes.

MMSE= Mini-Mental State Examination. RAVTL = Rey Auditory Verbal Learning Test. RCFT= Rey-Osterrieth Complex Figure Test. SDMT= Symbol Digit Modalities Test. CPT= Conner's Performance Task. IGT= Iowa Gambling Task.

2.13 Supplementary Table 13. Comparability of the population without type 2 diabetes (No-T2D) and the population with type 2 diabetes from subgroup 1 (T2D-S1) not treated with metformin before and after matching with inverse probability of treatment weights (IPTW)

		Unmatched						Matched				
		No-T2D		T2D-S1 No Metformin		D#	P-value	Rel Infl*	No-T2D	T2D-S1 No Metformin	D#	P-value
Variable	Category	N	% (SD) / Mean (SD)	N	% (SD) / Mean (SD)				% (SD) / Mean (SD)	% (SD) / Mean (SD)		
N		339	100%	36	100%							
Study group	Intervention group	162	47.8 (0.5)	20	55.6 (0.5)	0.155	0.376	0.5	47.9 (0.5)	55.8 (0.5)	0.158	0.380
Gender	Men	166	49 (0.5)	18	50 (0.5)	0.021	0.906	0.0	50.9 (0.5)	49.5 (0.5)	-0.029	0.871
Age	*Mean (SD)*	339	64.92 (4.7)	36	66.72 (5.2)	0.38	0.193	11.9	65 (4.7)	66.6 (5)	0.346	0.254
Education (years)	*Mean (SD)*	339	12.14 (5.7)	36	10.83 (4)	-0.233	0.519	5.4	12.1 (5.7)	10.9 (4)	-0.216	0.592
Current smoker		47	13.9 (0.3)	3	8.3 (0.3)	-0.163	0.355	0.5	13.9 (0.3)	7.7 (0.3)	-0.181	0.287
Intelligence Quotient	*Mean (SD)*	338	88.75 (39.5)	36	91.67 (44.4)	0.073	0.869	14.0	88.9 (39.6)	89.8 (43.5)	0.024	0.996
BMI (kg/m2)	*Mean (SD)*	339	32.37 (3.4)	36	32.23 (3.1)	-0.041	0.319	10.9	32.4 (3.4)	32.4 (3.2)	0.001	0.539
Waist circumference	*Mean (SD)*	339	106.77 (9.9)	36	106.66 (8.6)	-0.012	0.727	5.2	106.8 (9.9)	107.1 (8.6)	0.027	0.766
Total Cholesterol (mg/dL)	*Mean (SD)*	339	214.19 (39.3)	36	212.97 (40.3)	-0.031	0.309	1.5	214.1 (39.4)	212.3 (39.7)	-0.044	0.270
Triglycerides (mg/dL)	*Mean (SD)*	339	166.95 (100)	36	179.64 (80.9)	0.129	0.422	5.8	167.3 (99.7)	174.1 (79)	0.069	0.658
HDL-cholesterol (mg/dL)	*Mean (SD)*	339	52.37 (12.6)	36	48.03 (12.5)	-0.344	**0.028**	11.3	52.2 (12.6)	48.4 (12.3)	-0.304	0.115
LDL-cholesterol (mg/dL)	*Mean (SD)*	315	129.97 (33.3)	33	131.21 (37.6)	0.037	0.289	2.6	130 (33.4)	130.9 (37)	0.028	0.296
BDI-II total score	*Mean (SD)*	339	8.17 (6.7)	36	9.25 (7)	0.162	0.673	2.5	8.2 (6.6)	9.5 (6.9)	0.191	0.628
er-MedDiet adherence score	*Mean (SD)*	339	7.79 (2.5)	36	7.86 (2.4)	0.028	0.978	1.2	7.8 (2.5)	7.7 (2.4)	-0.021	0.960

Physical activity category	Sedentary	48	14.2 (0.3)	36	13.9 (0.3)	-0.008	0.886	1.3	14.3 (0.4)	14.9 (0.4)	0.017	0.911
	Under-active	238	70.2 (0.5)	5	66.7 (0.5)	-0.077			70.2 (0.5)	67.2 (0.5)	-0.065	
	Moderately active	25	7.4 (0.3)	24	11.1 (0.3)	0.14			7.3 (0.3)	10.6 (0.3)	0.122	
	Active	28	8.3 (0.3)	4	8.3 (0.3)	0.003			8.2 (0.3)	7.4 (0.3)	-0.032	
History of CVD grouped1		22	6.5 (0.2)	2	5.6 (0.2)	-0.038	0.828	0.0	6.7 (0.3)	5.8 (0.2)	-0.036	0.842
	Angina pectoris	1		1								
	Arrhythmia	21		2								
Other comorbidities grouped2		93	27.4 (0.4)	14	38.9 (0.5)	0.254	0.149	1.9	27.7 (0.4)	38.1 (0.5)	0.231	0.197
	Emphysema	13		1								
	Retinopathy	5		1								
	Nephropathy	7		0								
	Cholelithiasis	34		7								
	Gout	31		6								
	Cancer	18		1								
Depression		76	22.4 (0.4)	7	19.4 (0.4)	-0.072	0.683	0.0	22.4 (0.4)	20.1 (0.4)	-0.056	0.763
Sleep apnea		46	13.6 (0.3)	2	5.6 (0.2)	-0.24	0.173	1.2	13.4 (0.3)	4.7 (0.2)	-0.263	0.105
Medication for heart grouped3		52	15.3 (0.4)	7	19.4 (0.4)	0.113	0.521	0.0	15.5 (0.4)	19.2 (0.4)	0.100	0.572
	Medication for heart	21		2								
	Treatment with aspirin/Adiro®	33		5								
Medication for hypertension		251	74 (0.4)	28	77.8 (0.4)	0.086	0.626	0.0	74 (0.4)	76.1 (0.4)	0.049	0.789
Treatment for cholesterol		153	45.1 (0.5)	20	55.6 (0.5)	0.209	0.234	0.9	45.3 (0.5)	54.4 (0.5)	0.183	0.312
Tranquilizers/ sedatives		72	21.2 (0.4)	11	30.6 (0.5)	0.224	0.202	0.8	21.3 (0.4)	31.6 (0.5)	0.246	0.178

[1]Includes angina pectoris or arrhythmia

[2]Includes claudication, emphysema, retinopathy, nephropathy, cholelithiasis, gout and cancer

[3]Includes aspirin/Adiro®, other

*Relative influence of each variable for estimating the probability of treatment assignment.

Standardized difference statistic.

N= number.

2.14 Supplementary Table 14. Differences in MedDiet adherence and cognition at each time point and in the mean change from baseline between subjects without type 2 diabetes (No-T2D) and subjects with type 2 diabetes from subgroup 1 (T2D-S1, with 'Good-Stable' glycemic control trajectory) not treated with metformin (matched analyses with inverse probability of treatment weights)

Variable	Time	No-T2D [N=339][1]		T2D-S1 No Metformin [N=36][2]		Differences at each time point			Differences in the mean change (95%CI) from baseline		
		Missing [N (%)]	Mean (95%CI)	Missing [N (%)]	Mean (95%CI)	Cohen's D (95%CI)*	Effect Size[3]	P[4]	No-T2D	T2D-S1 No Metformin	P[5]
er-MedDiet adherence score	Baseline	0 (0.0)	7.8 (7.5, 8.1)	0 (0.0)	7.7 (6.9, 8.6)	-0.02 (-0.36, 0.32)	VS	0.906			
	1 year	12 (3.5)	11.7 (11.4, 12.1)	1 (2.8)	12.3 (11.2, 13.3)	0.17 (-0.17, 0.52)	S	0.348	4 (3.6, 4.3)	4.4 (3.5, 5.4)	0.408
	3 years	33 (9.7)	11.6 (11.3, 11.9)	1 (2.8)	12.6 (11.7, 13.4)	0.32 (-0.03, 0.67)	S	**0.046**	3.8 (3.4, 4.1)	4.7 (3.7, 5.8)	0.145
Memory composite (z-score)	Baseline	5 (1.5)	-0.02 (-0.09, 0.06)	1 (2.8)	-0.11 (-0.33, 0.12)	-0.13 (-0.48, 0.21)	VS	0.448			
	1 year	50 (14.7)	0.19 (0.11, 0.27)	7 (19.4)	0.13 (-0.14, 0.41)	-0.08 (-0.46, 0.3)	VS	0.686	0.19 (0.13, 0.25)	0.17 (-0.08, 0.43)	0.868
	3 years	83 (24.5)	0.56 (0.47, 0.65)	6 (16.7)	0.37 (0.08, 0.66)	-0.26 (-0.64, 0.12)	S	0.222	0.55 (0.47, 0.63)	0.36 (0.12, 0.6)	0.408
Executive functions composite (z-score)[1,2]	Baseline	41 (13.4)	-0.07 (-0.12, -0.01)	10 (34.5)	-0.12 (-0.35, 0.11)	-0.12 (-0.58, 0.35)	VS	0.649			
	1 year	74 (24.3)	0.01 (-0.05, 0.07)	10 (34.5)	-0.05 (-0.32, 0.21)	-0.12 (-0.59, 0.34)	VS	0.669	0.04 (-0.01, 0.08)	0.09 (0.01, 0.17)	0.479
	3 years	154 (50.5)	0.08 (0, 0.16)	14 (48.3)	0.27 (0.01, 0.54)	0.39 (-0.14, 0.92)	S	0.158	0.08 (0, 0.16)	0.33 (0.11, 0.55)	**0.032**
Global cognition composite (z-score)[1,2]	Baseline	43 (14.1)	-0.04 (-0.1, 0.02)	10 (34.5)	-0.09 (-0.27, 0.1)	-0.1 (-0.56, 0.37)	VS	0.639			
	1 year	78 (25.6)	0.1 (0.03, 0.16)	10 (34.5)	0.1 (-0.15, 0.36)	0.02 (-0.45, 0.48)	VS	0.954	0.12 (0.08, 0.16)	0.14 (-0.03, 0.3)	0.694
	3 years	155 (50.8)	0.3 (0.22, 0.37)	14 (48.3)	0.4 (0.14, 0.66)	0.23 (-0.3, 0.77)	S	0.430	0.25 (0.18, 0.32)	0.28 (0.07, 0.5)	0.318
RAVTL: immediate recall	Baseline	0 (0.0)	7.6 (7.3, 7.8)	0 (0.0)	7.1 (6, 8.2)	-0.14 (-0.49, 0.2)	VS	0.476			
	1 year	45 (13.3)	8 (7.6, 8.3)	6 (16.7)	7.3 (6.1, 8.4)	-0.24 (-0.62, 0.13)	S	0.236	0.4 (0.1, 0.7)	0 (-1, 1.1)	0.377
	3 years	81 (23.9)	9.4 (9, 9.8)	6 (16.7)	8.3 (7.5, 9.2)	-0.34 (-0.72, 0.04)	S	**0.027**	1.8 (1.4, 2.2)	1.2 (0.2, 2.1)	0.287
RAVTL: delayed recall	Baseline	0 (0.0)	7.4 (7.1, 7.7)	0 (0.0)	7 (6.1, 7.9)	-0.12 (-0.47, 0.22)	VS	0.469			

	1 year	45 (13.3)	7.7 (7.3, 8)	6 (16.7)	7.8 (6.6, 9.1)	0.04 (-0.33, 0.42)	VS	0.839	0.4 (0.1, 0.7)	0.8 (-0.3, 1.9)	0.482
	3 years	81 (23.9)	9.4 (9, 9.8)	6 (16.7)	8.2 (7.2, 9.1)	-0.38 (-0.75, 0)	S	**0.015**	2 (1.6, 2.4)	1.2 (0, 2.3)	0.213
RCFT: immediate recall	Baseline	4 (1.2)	13.9 (13.1, 14.6)	1 (2.8)	14.8 (12.3, 17.2)	0.13 (-0.22, 0.48)	VS	0.490			
	1 year	47 (13.9)	15.4 (14.6, 16.2)	7 (19.4)	16.2 (13.4, 19)	0.11 (-0.27, 0.49)	VS	0.581	1.1 (0.5, 1.7)	0.5 (-1.3, 2.4)	0.524
	3 years	81 (23.9)	17 (16.2, 17.8)	6 (16.7)	17.9 (14.9, 20.8)	0.12 (-0.25, 0.5)	VS	0.581	2.9 (2.1, 3.6)	1.9 (-0.3, 4)	0.857
RCFT: delayed recall	Baseline	5 (1.5)	13.6 (12.9, 14.3)	1 (2.8)	13.7 (11.5, 15.9)	0.01 (-0.33, 0.36)	VS	0.939			
	1 year	50 (14.7)	15 (14.2, 15.8)	7 (19.4)	16 (13.5, 18.5)	0.14 (-0.24, 0.52)	VS	0.460	1.2 (0.6, 1.7)	1.6 (-0.4, 3.6)	0.610
	3 years	83 (24.5)	17.2 (16.3, 18)	6 (16.7)	17.3 (14.3, 20.4)	0.02 (-0.35, 0.4)	VS	0.915	3.4 (2.6, 4.1)	2.6 (0.5, 4.7)	0.730
RCFT: recognition	Baseline	0 (0.0)	19.2 (19, 19.5)	0 (0.0)	18.6 (17.7, 19.5)	-0.29 (-0.63, 0.06)	S	0.152			
	1 year	45 (13.3)	20.2 (19.9, 20.4)	6 (16.7)	19.5 (18.3, 20.7)	-0.3 (-0.68, 0.07)	S	0.293	0.9 (0.6, 1.2)	0.9 (-0.2, 2)	0.810
	3 years	81 (23.9)	20.5 (20.2, 20.7)	6 (16.7)	20 (19.2, 20.8)	-0.21 (-0.59, 0.17)	S	0.313	1.1 (0.8, 1.4)	1.3 (0.1, 2.4)	0.959
RCFT: Copy	Baseline	4 (1.2)	26.8 (26, 27.6)	1 (2.8)	27.3 (24.7, 29.9)	0.06 (-0.29, 0.41)	VS	0.731			
	1 year	45 (13.3)	27.4 (26.6, 28.3)	7 (19.4)	27.2 (24, 30.5)	-0.02 (-0.41, 0.36)	VS	0.913	0.3 (-0.3, 0.9)	0.3 (-2, 2.5)	0.853
	3 years	81 (23.9)	29.5 (28.8, 30.2)	6 (16.7)	29.4 (26.9, 31.8)	-0.03 (-0.4, 0.35)	VS	0.912	2.8 (2, 3.6)	1 (-0.9, 3)	0.433
SMDT: direct	Baseline	25 (7.4)	36.7 (35.3, 38.1)	7 (19.4)	35.2 (30, 40.4)	-0.12 (-0.5, 0.26)	VS	0.569			
	1 year	51 (15)	36 (34.6, 37.4)	7 (19.4)	33.1 (27.5, 38.8)	-0.23 (-0.61, 0.16)	S	0.333	-0.9 (-1.8, 0)	-1.5 (-4.3, 1.2)	0.644
	3 years	81 (23.9)	34.2 (32.7, 35.6)	6 (16.7)	33.4 (28, 38.9)	-0.06 (-0.44, 0.32)	VS	0.795	-2.5 (-3.5, -1.4)	-1.5 (-4.9, 1.9)	0.604
Stroop: Interference	Baseline	0 (0.0)	-1.3 (-2.3, -0.3)	0 (0.0)	1.2 (-2.1, 4.4)	0.26 (-0.08, 0.61)	S	0.159			
	1 year	45 (13.3)	-1.1 (-2, -0.1)	6 (16.7)	2.1 (-1.2, 5.5)	0.39 (0.01, 0.77)	S	0.072	0 (-1, 1)	0.4 (-3.3, 4.1)	0.569
	3 years	81 (23.9)	-1.1 (-2.1, -0.2)	6 (16.7)	-1.2 (-5.1, 2.7)	-0.01 (-0.39, 0.37)	VS	0.971	-0.4 (-1.7, 0.8)	-3.7 (-8.3, 0.9)	0.601
IGT: total	Baseline	13 (4.3)	1.7 (-0.8, 4.1)	1 (3.4)	-6.3 (-12.6, -0.1)	-0.38 (-0.77, 0.01)	S	**0.019**			
	1 year	62 (20.3)	3 (0.2, 5.9)	8 (27.6)	-6.2 (-14.2, 1.7)	-0.41 (-0.86, 0.04)	S	**0.031**	0.6 (-3, 4.2)	-1.4 (-12.6, 9.7)	0.745
	3 years	142 (46.6)	7.3 (3.8, 10.7)	12 (41.4)	7.5 (-8, 23)	0.01 (-0.49, 0.51)	VS	0.979	4.5 (-0.1, 9.2)	16.8 (-1.5, 35.1)	0.171

CPT: Omission errors	Baseline	5 (1.6)	7.3 (5.6, 9)	2 (6.9)	8 (1.3, 14.7)	0.05 (-0.35, 0.44)	VS	0.843			
	1 year	57 (18.7)	5.7 (4.4, 7)	7 (24.1)	8.1 (-0.5, 16.7)	0.21 (-0.23, 0.64)	S	0.583	-1.3 (-2.9, 0.3)	1.4 (-1.3, 4)	0.676
	3 years	145 (47.5)	7.9 (4.1, 11.7)	12 (41.4)	4.7 (1.7, 7.6)	-0.14 (-0.64, 0.36)	VS	0.192	1.4 (-2.4, 5.2)	-3.5 (-12.6, 5.7)	0.423
CPT: Commission errors	Baseline	5 (1.6)	23 (21.2, 24.8)	2 (6.9)	20.3 (14.6, 25.9)	-0.17 (-0.56, 0.23)	S	0.370			
	1 year	57 (18.7)	20.4 (18.6, 22.3)	7 (24.1)	19 (13, 25)	-0.1 (-0.53, 0.34)	VS	0.646	-2 (-3.4, -0.6)	-1.5 (-6.1, 3.1)	0.839
	3 years	145 (47.5)	17.3 (15.3, 19.3)	12 (41.4)	14.1 (9.2, 19)	-0.25 (-0.75, 0.26)	S	0.239	-3.4 (-5.3, -1.5)	-1.6 (-5.5, 2.3)	0.583
CPT: HRT	Baseline	6 (2)	457.8 (449.1, 466.4)	2 (6.9)	485.9 (448.9, 523)	0.36 (-0.04, 0.75)	S	0.143			
	1 year	59 (19.3)	459 (450.1, 468)	7 (24.1)	473.7 (446.4, 501)	0.21 (-0.23, 0.64)	S	0.311	3 (-5.9, 11.9)	12.4 (-12.1, 36.8)	0.922
	3 years	145 (47.5)	454.9 (440.9, 468.9)	12 (41.4)	448.1 (413.9, 482.3)	-0.08 (-0.58, 0.42)	VS	0.715	-0.3 (-12.7, 12.2)	-10.3 (-56.2, 35.5)	0.186
MMSE: total score	Baseline	5 (1.5)	28.7 (28.5, 28.9)	0 (0.0)	28.3 (27.6, 28.9)	-0.24 (-0.59, 0.1)	S	0.238			

[1]N=309 and [2]N=29 when excluding participants from University of Valencia study site that did not perform the CPT and IGT.

[3]Effect Size: VS= very small (Cohen’s d <0.2); S= small (Cohen’s d (0.2-0.5)); M= medium (Cohen’s d (0.5-0.8)); L= large (Cohen’s d (0.8-1.2)); VL= very large (Cohen’s d ≥ 1.2).

[4]ANOVA from multivariable-adjusted linear model

[5]ANOVA from multivariable-adjusted linear mixed effects model.

Inverse probability of treatment weighting (IPTW) was applied to all analyses to weight each individual with his/her inverse probability of being treated with metformin, generating a pseudo-population with (almost) perfect covariate balance.

MMSE= Mini-Mental State Examination. RAVTL = Rey Auditory Verbal Learning Test. RCFT= Rey-Osterrieth Complex Figure Test. SDMT= Symbol Digit Modalities Test. CPT= Conner's Performance Task. IGT= Iowa Gambling Task.

*Reference group= No-T2D

3 Supplementary Figures

3.1 Supplementary Figure 1. Description of metformin treatment maintenance in subjects with type 2 diabetes from subgroup 1 (‘Good Stable glycemic control pattern’, N=123).

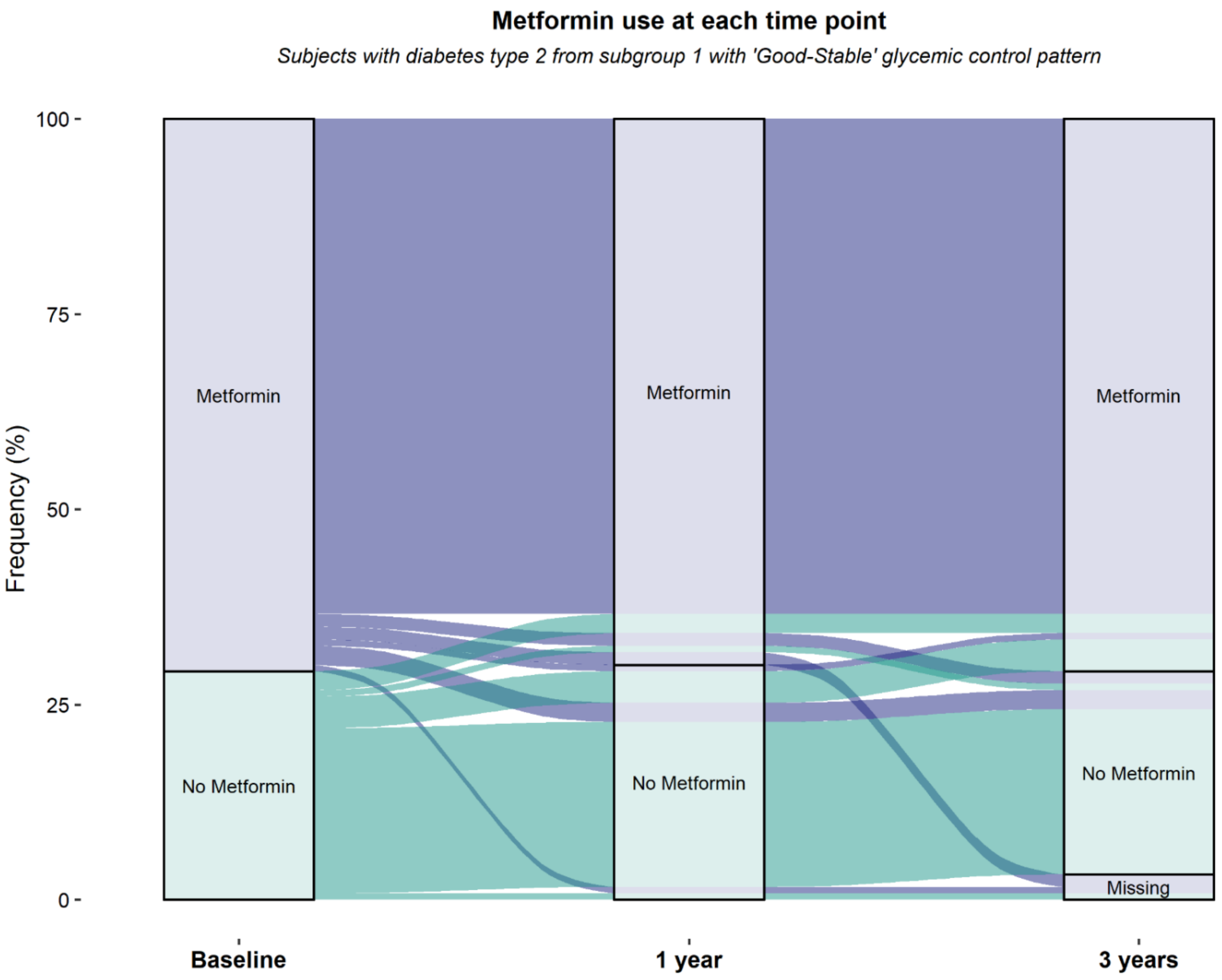

Supplement: Supplementary file 1 [file Data_Sheet_1.PDF]
