# Supplementary figures and images for: Metformin Use and Cognitive Function in Older Adults With Type 2 Diabetes Following a Mediterranean Diet Intervention
Source: Front Nutr. 2021 Oct 5;8:742586. doi: 10.3389/fnut.2021.742586 (PMC8523839; doi:10.3389/fnut.2021.742586)

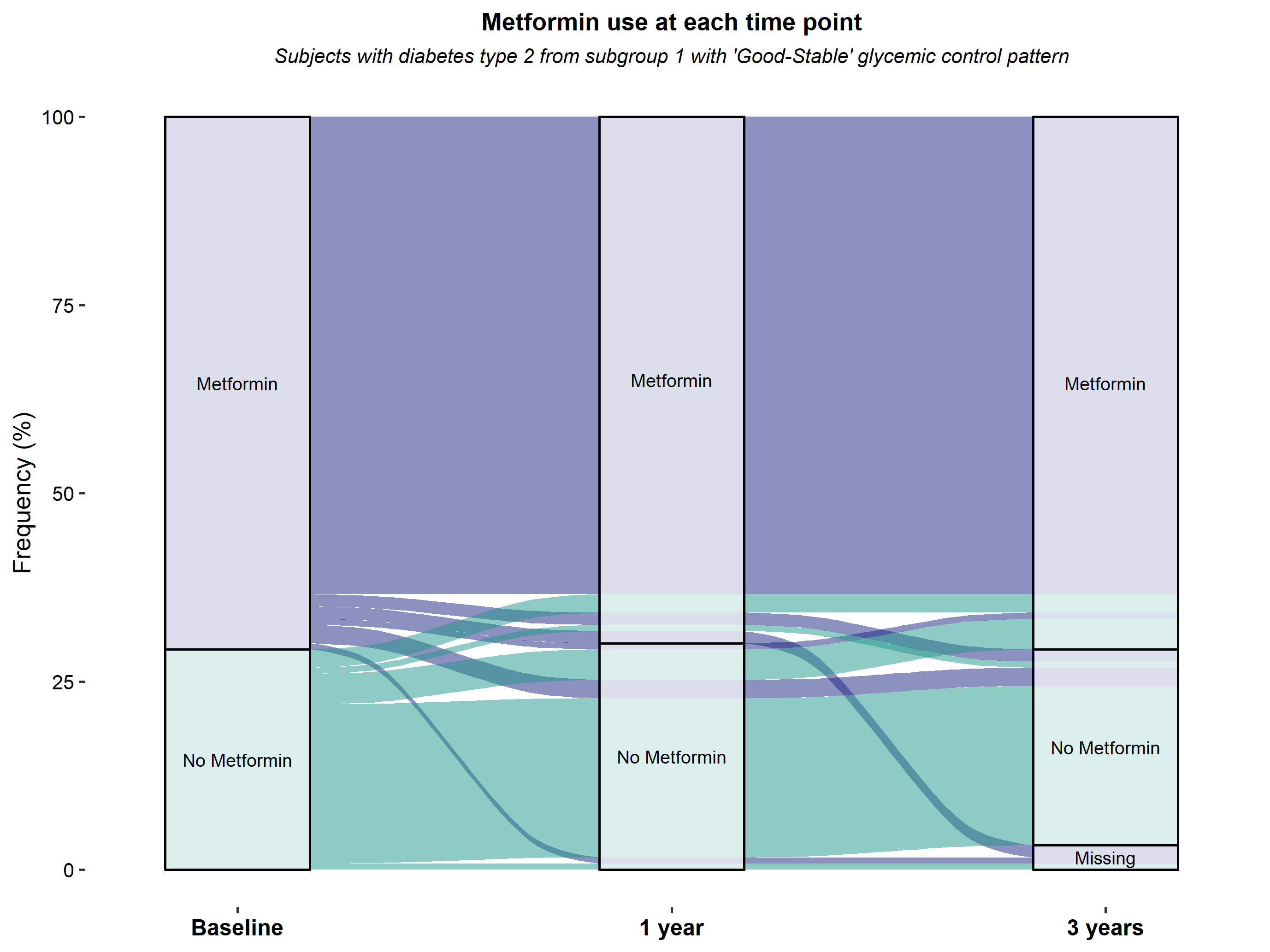

Supplement: Supplementary file 2 [file Image_1.TIFF]
